# CD147: a small molecule transporter ancillary protein at the crossroad of multiple hallmarks of cancer and metabolic reprogramming

**DOI:** 10.18632/oncotarget.14272

**Published:** 2016-12-27

**Authors:** Agnieszka A. Kendrick, Johnathon Schafer, Monika Dzieciatkowska, Travis Nemkov, Angelo D'Alessandro, Deepika Neelakantan, Heide L. Ford, Chad G. Pearson, Colin D. Weekes, Kirk C. Hansen, Elan Z. Eisenmesser

**Affiliations:** ^1^ Department of Biochemistry and Molecular Genetics, School of Medicine, University of Colorado Denver, CO, USA; ^2^ Department of Pharmacology, School of Medicine, University of Colorado Denver, CO, USA; ^3^ Department of Cell and Developmental Biology, School of Medicine, University of Colorado Denver, CO, USA; ^4^ Division of Oncology, Department of Medicine, University of Colorado Denver, CO, USA

**Keywords:** metabolism, ancillary protein, PDAC, transmembrane, tumor microenvironment

## Abstract

Increased expression of CD147 in pancreatic cancer has been proposed to play a critical role in cancer progression via CD147 chaperone function for lactate monocarboxylate transporters (MCTs). Here, we show for the first time that CD147 interacts with membrane transporters beyond MCTs and exhibits a protective role for several of its interacting partners. CD147 prevents its interacting partner's proteasome-dependent degradation and incorrect plasma membrane localization through the CD147 transmembrane (TM) region. The interactions with transmembrane small molecule and ion transporters identified here indicate a central role of CD147 in pancreatic cancer metabolic reprogramming, particularly with respect to amino acid anabolism and calcium signaling. Importantly, CD147 genetic ablation prevents pancreatic cancer cell proliferation and tumor growth *in vitro* and *in vivo* in conjunction with metabolic rewiring towards amino acid anabolism, thus paving the way for future combined pharmacological treatments.

## INTRODUCTION

Pancreatic ductal adenocarcinoma (PDAC), the predominant form of pancreatic cancer, is the fourth leading cause of cancer related deaths in the United States with a 5-year survival rate of less than 6% [1]. It is well established that over 90% of PDACs exhibit activated KRAS mutations, which drive pancreatic neoplasia; however, therapeutically targeting KRAS has proven widely unsuccessful [2]. One of the biggest challenges in targeting PDAC progression is the high level of stromal content in the tumor volume, which creates a highly hypoxic and acidic microenvironment that leads to the metabolic reprogramming of tumor cells [3]. Thus, targeting the specific metabolic changes associated with PDAC progression is an attractive therapeutic approach.

CD147 (also known as EMMPRIN or basigin) is a highly glycosylated type I single pass transmembrane protein upregulated in a variety of cancers including PDAC [4]. Silencing of full length CD147 in PDAC leads to inhibition of malignant potential and cell invasion [5]. CD147 was initially characterized as a matrix metalloproteinase (MMPs) inducer, although such studies have recently been met with controversy. Specifically, Marchiq et al. presented evidence for CD147-mediated regulation of fermentative glycolysis without any involvement in MMPs induction [6]. Several other functions of CD147 have since been portrayed, including the regulation of cell proliferation, Wnt signaling, and the epithelial to mesenchymal transition (EMT or mesenchymal to epithelial transition - MET) [7]. Though, in each case the specific mechanistic details of CD147 function have not been fully characterized. Moreover, CD147 extracellular region contains three different sites of glycosylation (N44, N152 and N186), although there is conflicting evidence in regard to the involvement of CD147 glycosylation in its activity and interactions [8, 9].

One explanation regarding the role of CD147 in cancer metastasis and proliferation is its interaction with monocarboxylate transporters (MCTs) [10]. Studies on Warburg-like glycolytic reprogramming as a key hallmark of (pancreatic) cancer have fostered our understanding of MCTs [11]. MCTs are multi-pass transmembrane proteins that are responsible for transport of small molecules (e.g. lactate) across plasma membrane [12], and thus, the regulation of cellular metabolic processes. MCTs are widely expressed in several tissues, and the hypoxia-inducible member MCT4 in particular is highly expressed in glycolytic malignant tumors, which include PDAC cells [13]. Recent discoveries indicate that CD147 may play a chaperone role for MCT1 and MCT4. Marchiq et al. showed in colon cancer and glioblastoma cells that genetic ablation or pharmacological inhibition of MCT/CD147 complex had a detrimental effect on cancer progression [14]. However, the role of CD147/MCT interactions in regard to PDAC progression has not been determined [10, 15]. Interestingly, depletion of MCT4 in PDAC cells diminishes cell growth and viability, while CD147 depletion in these same cells affects tumor growth through an alternative mechanism that has yet to be characterized [16]. Based on these observations, we hypothesized that although CD147 can function cooperatively with MCTs, there are likely other factors that underlie CD147 function. Thus, the focus of this study was to identify these factors and characterize the role of CD147 function in PDAC.

## RESULTS

### CD147 regulates cell proliferation, adhesion and migration in a mechanism only partially dependent on MCTs

**Figure 1:**
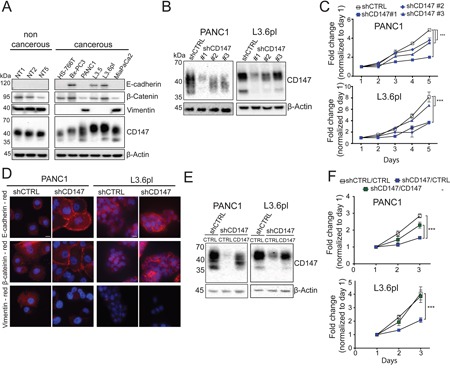
CD147 downregulation inhibits cancer phenotypes **A.** CD147 and EMT marker proteins are expressed in pancreatic cell lines. β-actin provided a loading control. **B.** Stable CD147 knockdown in pancreatic cancer cell lines. β-actin provided a loading control. **C.** CD147 knockdown decreases cell proliferation. Cell proliferation was measured by tryptan blue staining and daily cell counts. All cell counts were normalized to day 1 for the indicated cell lines and reported as fold changes (*^,^p < 0.05, **p < 0.01, ***p < 0.001). **D.** CD147 reverses the expression of multiple markers associated with the EMT. PANC1 cells were stained with CD147, E-cadherin, β-catenin and Vimentin antibodies and representative images are shown. Cell nuclei were counterstained with Hoechst (blue). Scale bar, 10 μm. **E.** Rescue of CD147 expression reinstates CD147 levels. shCD147#1 was transduced with control or CD147 lentivirus. β-actin provided a loading control. **F.** CD147 re-expression rescues cell growth. All cell counts were normalized to day 1 for the indicated cell lines and reported as fold changes (*^,^p < 0.05, **p < 0.01, ***p < 0.001). Representative results are shown.

CD147 is highly expressed in most pancreatic cells; however, cancer cell lines express higher molecular weight CD147 (35-65 kDa) compared to the non-cancerous cells (35-45 kDa) indicating increased levels of glycosylation (Figure 1A). Based on the diverse expression of epithelial markers in PDAC cells (Figure 1A) and previous evidence for CD147 involvement in regulating the EMT/MET, we selected mesenchymal (PANC1) and epithelial (L3.6pl) cells or further analysis. Initially we attempted to generate a CD147 knock-out cells using CRISPR/Cas9 system, however, we were not able to isolate viable cells harboring a complete depletion of CD147 (data not shown). Although, such attempts have previously been reported in some cancer systems, the lack of success in our case could potentially be explained by the importance of CD147 in PDAC progression [6, 14]. Using three different shRNAs, each targeting different region of the CD147 transcript, we generated stable knockdown cells with varying levels of CD147 downregulation (Figure 1B). Of the three knockdowns, shCD147#1 generated the most robust decrease in CD147 expression and strongest phenotypic changes. shCD147#3 exhibited strong initial diminishment in CD147 expression as well, though this trait was lost over time and lead to more variability in the observed phenotypes (Supplementary Figure 1A). For each CD147 knockdown cell line, we observed corresponding attenuation of cell migration and growth (Figure 1C and Supplementary Figure 1B), followed by the MET appearance (Figure 1D and Supplementary Figure 1C). As expected, L3.6pl cells showed no significant changes in the EMT or migration (Figure 1D and Supplementary Figure 1B–1C) due to their initial epithelial characteristics (Figure 1A). To further confirm the specificity of CD147 depletion, we generated CD147 rescue cell lines using the knockdown cells with the most significant downregulation of CD147 (shCD147#1). As expected, re-introduction of a CD147 construct into CD147 depleted cells (Figure 1E) was accompanied by an increase in cell growth (Figure 1F) and the restoration of the EMT (Supplementary Figure 1D). These observations suggest that CD147 plays a critical role in regulating PDAC progression via regulation of the EMT and are consistent with other studies that illustrate the involvement of CD147 in regulating cancerous phenotypes [15].

### CD147-mediated metabolic regulation of PDAC progression relies on MCTs and other factors

**Figure 2:**
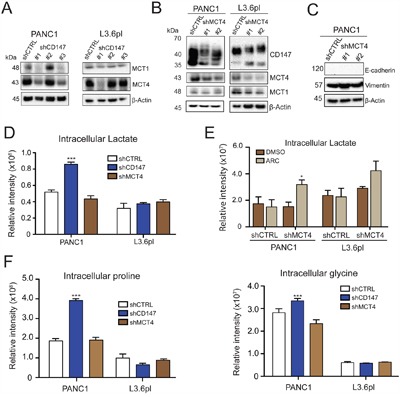
CD147 function is only partially dependent on MCTs **A.** CD147 knockdown leads to decreased levels of MCT1 and MCT4. β-actin provided a loading control. **B.** MCT4 depletion leads to modest CD147 downregulation, but does not significantly affect MCT1 levels. Indicated cell lines were stably transduced with lentiviral particles encoding scramble control (shCTRL) or shRNA targeting MCT4 gene. β-actin provided a loading control. **C.** MCT4 knockdown does not impart the same effects on Vimentin and E-cadherin expression as CD147 knockdown. β-actin provided a loading control. **D.** Intracellular lactate accumulates in CD147 knockdown PANC1 cells but not in MCT4 downregulated cells or L3.6pl cells. Metabolomics analysis for intracellular lactate levels in the indicated cell lines. Bars are ± SEM, n=3, ***p<0.001. **E.** The changes in intracellular lactate levels in MCT4 and MCT1 inhibited cells. The levels of intracellular lactate were measured in the PANC1 MCT4 knockdown cell line in the presence of absence of MCT1 inhibitor (ARC). Bars are ± SEM, n=3, *p<0.05. **F.** CD147 knockdown cells exhibit metabolic profile distinct from MCT4 depleted cells. Metabolomics analysis for designated intracellular amino acids. Bars are ± SEM, n=3, ***p<0.001.

To determine if the specific mechanism of CD147 function in PDAC was based on interactions with MCTs, we tested the contribution of MCT1 and MCT4 to the phenotypes we identified above above. Importantly, both MCT1 and MCT4 were downregulated in CD147 depleted cells (Figure 2A and quantification in Supplementary Figure 2A) suggesting a functional relationship between these proteins. As expected, MCT4 depletion was reversed by restoration of CD147 in the CD147 rescue cells (Supplementary Figure 2B). We next tested the specific effects of MCT1 and MCT4 inhibition in CD147 knockdown cells. MCT4 stable depletion (Figure 2B) led to a significant reduction in CD147 levels and a substantial decline in cell growth, with no effect on MCT1 levels (Supplementary Figure 2C). Consistent with previously published results in PDAC cells [17], we also observed no variation in the levels of the EMT markers in PANC1 cells (Figure 2C) thus indicating that MCT4 is not involved in the EMT regulation. This result was also replicated in MCT4 depleted cells treated with the MCT1 inhibitor ARC-C155858(referred to herein as ARC) that recapitulates a phenotype in which both MCT1 and MCT4 activities/expression are inhibited, as we hypothesized would be the case in CD147 depleted cells (Figure 2A). We chose this approach based on similar studies that used MCT1 inhibitors to decouple the combined effect of MCT4 and MCT1 activity inhibition from CD147 function [14]. Although MCT4 pharmacological inhibitors are not available, MCT1 can easily be inhibited pharmacologically without affecting the MCT1 protein level. ARC specifically inhibits MCT1 and MCT2 without affecting MCT4 activity [18], although MCT2 is a high affinity pyruvate transporter expressed at only low levels in the pancreas [19]. MCT4 depleted cells treated with the MCT1 inhibitor displayed no changes in the expression of EMT markers (Supplementary Figure 2D), supporting the supposition that although MCT1 and MCT4 are regulated by CD147, there are clearly other functions that underlie CD147 activity in the context of EMT.

Because MCT1 and MCT4 are proton-coupled lactate transporters, decreased expression of these proteins that occurs in response to reduced CD147 expression should be accompanied by intracellular lactate accumulation. Thus, we investigated the functional consequences of CD147 knockdown and subsequent MCT4 and MCT1 depletion by measuring cellular lactate levels through an LC-MS based metabolomics analysis of cellular extracts. We found significant accumulation of intracellular lactate in CD147 depleted PANC1 cells with insignificant changes to lactate levels in CD147 depleted L3.6pl cells (Figure 2D), which are in agreement with the extent of CD147 mediated phenotypic changes observed in these cells. In addition, MCT4 depletion did not lead to a significant accumulation of intracellular lactate (Figure 2D), likely due to a compensatory activity of MCT1. We tested this possibility by treating MCT4 knockdown cells with ARC. As shown in Figure 2E, MCT4 depleted cells treated with ARC exhibited accumulation of intracellular lactate. Furthermore, CD147 restoration yield a decrease in intracellular lactate levels compared to the initial CD147 knockdown (Supplementary Figure 2E). Interestingly, we detected an overall increase in intracellular amino acids in CD147 depleted PANC1 cells relative to the other tested conditions (Figure 2F and Supplementary Figure 2F). Our observations that MCT1 and MCT4 inhibition do not result in similar phenotypic changes as CD147 depletion support the supposition that although MCT1 and MCT4 are regulated by CD147, additional functions may underlie the role of CD147 in regulating PDAC.

### CD147 depleted cells exhibit deregulation of key metabolic enzymes/transporters

**Figure 3:**
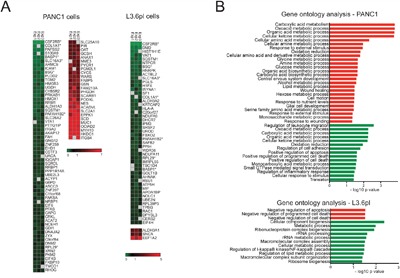
CD147 regulates adhesion and metabolic processes in CD147 depleted cells **A.** Proteomic changes due to CD147 knockdown identified by SILAC. Heat maps summarizing the protein abundance (log2) between three independent experiments for all statistically significant up- or down-regulated proteins. *Proteins common between both cell lines. Uniprot gene names were clustered using GENE-E software. **B.** Classification of proteomic changes monitored via SILAC. Graphs representing Gene Ontology (GO) analysis for all statistically significant up- or down-regulated proteins. Only terms with a p-value < 0.05 were selected.

The striking influence of CD147 downregulation on cell growth and migration led us to investigate the global proteome changes upon CD147 depletion in PDAC cells via stable isotope labeling by amino acid in cell culture (SILAC). In order to better represent the overall role of CD147 in PDAC regulation, we used cells harboring the most significant CD147 depletion from two cell types, shCD147#1 PANC1 and shCD147#1 L3.6pl. We identified a total of 87 and 51 proteins differentially regulated in PANC1 and L3.6pl cells, respectively (Figure 3A and Supplementary Figure 3A). Out of these groups, a significantly larger number of proteins were downregulated rather than upregulated, which expectedly included CD147 and its proposed interacting partner, MCT4. Consistent with the extent of phenotypic changes observed in L3.6pl cells relative to PANC1 cells, L3.6pl cells had a fewer number of differentially expressed proteins with 7 proteins present in both PANC1 and L3.6pl cells (Figure 3A, marked with a star).

Gene ontology (GO) term enrichment revealed that the vast majority of the proteins that are quantitatively affected by CD147 knockdown are implicated in the regulation of adhesion, cell motion and metabolic processes (Figure 3B), which is consistent with the observed MET (Figure 1D). Specifically, transglutaminase 2 (TGM2) and aldehyde dehydrogenase 1 A3 (ALDH1A3, gene name: ALDH1A3), which are mesenchymal markers, were decreased in CD147 depleted cells (Figure 3A and Supplementary Figure 3B) [20, 21]. Decreased levels of integrin β4 (gene name ITGB4) in CD147 depleted cells (Figure 3A and Supplementary Figure 3C) has been recently shown to promote the EMT in PDAC [22]. Importantly, CD147 knockdown cells showed differential levels of multiple metabolic proteins in addition to proteins involved in cellular adhesion and motility. In particular, pyruvate kinase isoenzyme 2 (PKM2) and glucose-6-phosphate dehydrogenase (G6PD), enzymes involved in glycolysis and pentose phosphate pathway (PPP), respectively, were both downregulated in CD147 depleted cells (Figure 3A and Supplementary Figure 3B). Other proteins critical for the activation of glycolysis were upregulated with CD147 depletion, including glucose transporter 1 (GLUT1, gene name SLC2A1) and hexokinase containing domain 1 (HKDC1), one of the isoforms of hexokinase (Figure 3A). Enzymes involved in serine, proline and arginine biosynthesis including phosphoglycerate dehydrogenase (PHGDH), ornithine aminotransferase, (OAT) and pyrroline-5-carboxylate reductase 1 (PYCR1), were all upregulated (Figure 3A). Thus, knockdown of a single protein, CD147, culminates in a substantial global proteome change that is consistent with the phenotypic changes in cellular metabolic reprograming and the MET as described above.

### CD147 associates with several transmembrane proteins beyond its interactions with MCTs

**Figure 4:**
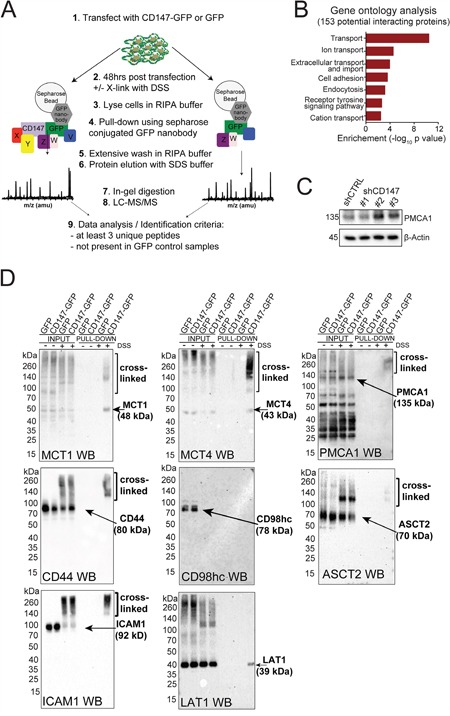
CD147 interacts with multiple transmembrane proteins beyond its interaction with MCTs **A.** Schematic representation of the cross-linking/pull-down strategy utilized here for the identification of CD147 interacting partners through MS analysis. **B.** Classification of CD147 interactions monitored via a cross-linking/MS approach. GO terms analysis for all 153 proteins identified. Only terms with a p-value < 0.05 are listed. Western blot analysis further validates CD147 interacting partners identified via cross-linking/MS. Arrows indicate the location of the correct molecular weight for each identified proteins. Cross-linked fractions are also marked. **C.** CD147 knockdown leads to downregulation of PMCA1. β-actin provided a loading control. **D.** Western blot validation of CD147-specific interactions identified via cross-linking/pull-downs. Arrows indicate the location of the correct molecular weight bands for each identified proteins. Cross-linked fractions are also marked.

Given the appreciated role of CD147 as an ancillary protein to MCTs [10], we hypothesized that CD147 expression can influence other metabolic enzymes/transporters via chaperone-like interactions. Using GFP-nanobody pull-down/cross-linking followed by LC-MS/MS (Figure 4A and Supplementary Figure 4A), we identified 153 proteins specifically enriched in the CD147-GFP fractions (Supplementary Table 1). A majority of these proteins, which include MCT4, are involved in cellular transport of small molecule metabolites (Figure 4B). In addition, we identified 18 proteins involved in the regulation of cell adhesion, consistent with the functional analysis of our global MS data and thus suggesting a link between CD147 interactome and CD147 signaling.

**Figure 5:**
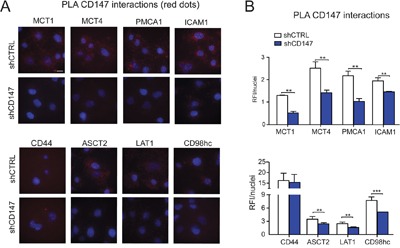
Endogenous CD147 interactions are further confirmed using PLA assay **A.** Proximity ligation assay (PLA) confirms endogenous CD147 interactions. Interactions (< 40nm) of CD147 with target proteins are indicated as red dots. Cell nuclei were counterstained with Hoechst (blue). Scale bar 10μm. **B.** Most CD147 interactions are significantly downregulated in CD147 depleted cells. RFI (relative fluorescence intensity) of images was quantified and normalized to nuclei number. Bars are ± SEM, **p<0.01.

To validate potential CD147 interactions, we focused on proteins that were identified as having a high likelihood of association with CD147 based on enrichment in CD147-GFP samples as well as previous suggestion of such interactions [23]. For these studies, we selected MCT1, MCT4, plasma membrane calcium ATPase 1 (PMCA1), alanine/serine/cysteine transporter 2 (ASCT2), L-system amino acid transporter (LAT1), intracellular adhesion molecule 1 (ICAM1), CD44 and CD98hc. Interestingly, several of the identified proteins (MCT4, MCT1, PMCA1, CD44 and ICAM1) were highly downregulated (Figure 2A, 4C and Supplementary Figure 4B) while other proteins (ASCT2, LAT1 and CD98hc) were upregulated in CD147 depleted cells (Supplementary Figure 4B). We confirmed several CD147 cross-linked complexes and endogenous interactions via immunoblotting and an *in situ* proximity ligation assay (PLA, Figure 4D and Figure 5A-5B). As expected, these interactions were diminished in CD147 depleted cells (Figure 5A–5B shCD147 cells). We did not observe any signal in the cross-linked samples (input or pull-down) for CD98hc (Figure 4D), but did confirm that LAT1, the CD98hc ancillary protein, is pulled down within a CD147 complex (Figure 4D). Since CD98hc forms a complex with LAT1, this may imply that the antibody epitope to CD98hc was lost due to cross-linking or that a LAT1/CD147 interaction is mediated by another protein [24]. We did, however, test the interaction of the recombinantly purified CD98hc ectodomain (CD98hc-ECD) with the CD147-ECD through atomic resolution studies (Supplementary Figure 7A), since such an association has been previously suggested [23]. Using chemical shift perturbations analysis (Supplementary Figure 7A), we observed no interaction between the CD147-ECD and CD98hc-ECD, indicating that the CD147/LAT1 complex is mediated through another region of CD98hc or another protein.

### CD147 regulates cellular processes through its interaction with PMCA1

**Figure 6:**
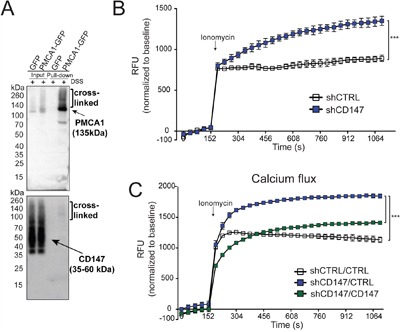
CD147 regulates cellular processes via its interaction with PMCA1 **A.** Pull-down of transfected PMCA1-GFP shows an association with CD147. Arrows indicate the location of the correct molecular weight band for identified proteins. Cross-linked fractions are also marked. **B.** CD147 knockdown leads to the accumulation of cellular calcium. Indicated cells were loaded with Fluoro-4 for 1 hr at 37°C followed by stimulation with 1 uM ionomycin. Calcium response was measured over time and normalized to the baseline signal. **C.** Rescue of CD147 expression rescues MCT function of cellular lactate export. Metabolomics analysis of intracellular lactate levels. See Methods for experimental details. Bars are ± SEM, ***p<0.001.

We discovered that CD147 interacts with PMCA1, an ATP dependent calcium exporter critical for regulating calcium homeostasis [25]. Transfection of cells with PMCA1-GFP followed by cross-linking and pull-down and identified CD147 along with PMCA1 (Figure 6A and higher exposure in Supplementary Figure 5A). This is an important discovery since this interaction has not been previously described. We next assessed the functional consequences of CD147-PMCA1 engagement in PDAC cells by monitoring calcium flux response over time. Control cells with no aberrant PMCA1 levels extruded the intracellular calcium in a timely manner, while CD147 depleted cells exhibited increased intracellular calcium storage (Figure 6B). These data corroborate deregulated calcium efflux in CD147 knockdown cells that is consistent with the decreased expression of PMCA1 that can lead to deregulation of cellular processes important for cell maintenance and growth. Furthermore, stable re-introduction of CD147 construct into CD147 depleted cells restored calcium flux response (Figure 6C) and PMCA1 levels (Supplementary Figure 5B), both of which is indicate a re-establishment of PMCA1 activity.

### CD147 is an ancillary protein for its interacting partners predominantly through its transmembrane region

**Figure 7:**
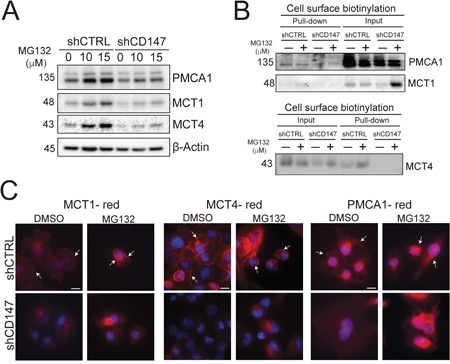
CD147 plays a chaperone role for its partner proteins **A.** Proteasomal inhibition partially rescues protein levels of CD147 target proteins. Indicated cells were treated with DMSO (0 μM MG132) or increasing concentrations of MG132 (proteasomal inhibitor). β-actin provided a loading control. **B.** The loss of CD147 in CD147 depleted cells is unable to localize its partner proteins to the cellular membrane, even when proteasomal degradation is inhibited. Cell surface biotinylation in the control or CD147 depleted cell lines treated with proteasomal inhibitor (MG 132) or DMSO control. Total protein (input) and membrane bound fractions (pull-down), were assessed by immunoblotting with the designated antibodies. **C.** Proteasomal inhibition rescues protein levels of CD147 partners, without restoring their cellular membrane. Immunofluorescence analysis of cells treated with DMSO or 10 μM MG132 for 6hrs following staining with antibodies specific for the identified proteins. Cell nuclei were counterstained with Hoechst (blue). Scale bar is 10 μm. Arrows point to cellular membranes.

Our findings illustrate that CD147 interacts with a number of membrane proteins and the expression of a subset of these proteins, including MCT1, MCT4 and PMCA1, is significantly suppressed upon CD147 depletion. This discovery led us to further investigate the specifics of these interactions and their functional consequences. CD147 was previously shown to be important for accompanying MCTs (MCT1 and MCT4) to the membrane to assure their proper cellular localization [26], thereby qualifying as a chaperone protein, which is defined as any protein that assists another protein in folding, translocation or protection against degradation [27]. Thus, to determine whether CD147 plays a chaperone role to its interacting partners in PDAC cells, we tested proposed chaperone functions such as translocation and protection from degradation. We treated control or CD147 depleted cells with increasing concentrations of a proteasomal inhibitor (MG132) and measured the changes in its interacting protein expression by Western blotting. Figure 7A illustrates that we were able to restore MCT1, MCT4 and PMCA1 expression when proteasomal degradation was inhibited, indicating that CD147 protects its interacting partners from degradation. Furthermore, we demonstrated by cell surface biotinylation (Figure 7B) and immunofluorescence (Figure 7C) that although inhibition of proteasomal degradation restores expression of CD147 interacting partners, these proteins are unable to translocate to the cellular membrane in the absence of CD147. This inability of the indicated proteins to translocate to the membrane in the absence of CD147 implies that CD147 is necessary for their proper translocation. Collectively, these data show that CD147 protects its targets from degradation and translocates them to the cellular membrane, indicating that CD147 is an ancillary protein for MCT1, MCT4 and PMCA1 in PDAC cells.

**Figure 8:**
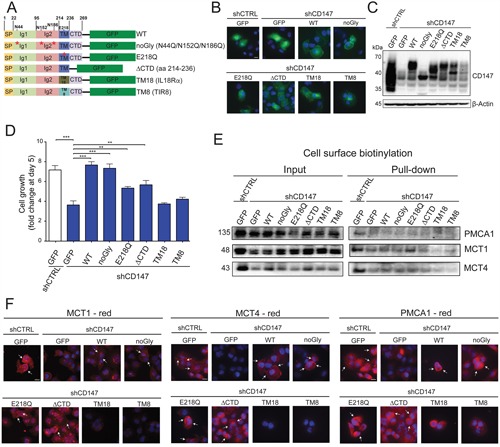
CD147 interactions are largely regulated through its transmembrane region **A.** Schematic representation of constructs generated to identify the regions of CD147 necessary for its interactions. WT - wild type, SP - signal peptide, TM - transmembrane region, CTD - C terminal domain, TM18 – TM region of IL-18Rα, TM8 – TM region of TIR8. **B.** GFP-tagged CD147 constructs are capable of localizing to the cellular membrane. Immunofluorescence of the indicated stable cell lines. Cell nuclei were counterstained with Hoechst (blue). Scale bar, 10 μm **C.** Expression of GFP-tagged CD147 constructs in CD147 knockdown cells. Indicated cell lines were stably transfected with GFP or GFP constructs outlined in C. β-actin provided a loading control. **D.** Cell growth is restored with re-expression of CD147 in CD147 depleted cells. Cell counts 5 days after plating. Bars are ± SEM, *^,^p < 0.05, **p < 0.01, ***p < 0.001. **E.** The cell-surface expression of CD147 partner proteins is dependent on different regions of CD147, as illustrated by cell surface biotinylation. Total protein (input) and membrane bound fractions (pull-down), were assessed by immunoblotting with the designated antibodies. **F.** The cell-surface expression of CD147 partner proteins is dependent on different regions of CD147. shCTRL or shCD147 PANC1 cells stably transfected with the indicated CD147-GFP construct were stained with antibodies specific for either MCT1, MCT4 or PMCA1. Cell nuclei were counterstained with Hoechst (blue). Scale bar, 10 μm.

To selectively disrupt CD147 interactions, we mapped the precise CD147 regions by mutagenesis analysis (Figure 8A). Specifically, we generated a glycosylation incompetent mutant (noGly), since glycosylation has been proposed to be important for CD147 cellular membrane localization [28]. We also mutated the conserved glutamic acid within the transmembrane region (E218Q), deleted the cytoplasmic tail (ΔCTD), and replaced the transmembrane region with transmembrane regions of two unrelated single pass transmembrane proteins (Single Ig IL-1-related receptor, TIR8 and Interleukin-18 receptor α, IL18Rα). In each case, we were able to restore the CD147 cellular membrane localization (Figure 8B) and at least partially restore CD147 expression (Figure 8C), consistent with the level of GFP signal observed in these cells (Supplementary Figure 6A). Furthermore, aside from CD147 transmembrane mutants (TM18 and TM8), all CD147 constructs were at least partially capable of rescuing cell growth due to initial CD147 depletion (Figure 8D and Supplementary Figure 6B), with the highest increase in cell growth for WT and noGly constructs and partial restoration for E218Q and ΔCTD. The reestablishment of the wild type and mutant CD147 expression was accompanied by restoring its interacting proteins levels (MCT4, MCT1 and PMCA1, Supplementary Figure 6C and Western quantification in S6D). Consistent with this functional analysis, the proper membrane localization of CD147 interacting partners relied primarily on the CD147 transmembrane region, as shown by cell surface biotinylation (MCT4, MCT1 and partially PMCA1, Figure 8E) and immunofluorescence (Figure 8F). This restoration of membrane expression effect was more robust for MCT1 and MCT4, as compared to PMCA1, possibly due to the more direct interaction of CD147 with MCTs [10].

Despite our findings that have identified the transmembrane region of CD147 as an important mediator of its activity in PDAC cells, the CD147 ECD has been proposed to comprise activity in other cell types and its role in PDAC cells remains unknown [29]. Thus, we tested the activity of CD147 extracellular region (glyCD147-ECD, aa 22-205, Supplementary Figure 7B) for the proposed MMP9 stimulation in PANC1 and L3.6pl cells, but detected no statistically relevant stimulatory activity (Supplementary Figure 7C). In addition, we tested the contribution of the CD147 ectodomain glycans to some of the previously proposed CD147 ectodomain interactions, including an interaction with the extracellular region of Syndecan-1 [30]. Utilizing NMR spectroscopy with isotopically labeled Syndecan-1, we detected no direct interaction between glyCD147-ECD (Supplementary Figure 7D). This lack of detected interaction with the extracellular region of Syndecan-1 suggests that CD147 ECD does not comprise the primary determinant of CD147 activity in PDAC cells and is consistent with our findings that show the full length CD147 responsible for its functions.

### CD147 interactions mediate growth inhibition via metabolic reprogramming of amino acid metabolism

Therapeutically targeting metabolic pathways has become a very attractive strategy directed at halting cancer progression. Here we have shown that CD147 affects the stability and membrane localization of key small molecule transporters, including, but not limited to, MCT lactate transporters. Therefore, we hypothesized that CD147 deletion would promote metabolic reprogramming beyond intracellular accumulation of glycolysis-derived lactate alone. We thus incubated PDAC cells with stable isotope tracers *in vitro*, either ^13^C_6_-glucose or ^13^C_6_^15^N_2_-glutamine, to assess the metabolic fluxes of glucose and glutamine oxidation by monitoring the incorporation of the heavy carbon and nitrogen atoms into downstream metabolites. Labeling experiments were performed in PANC1 cells since cell proliferation was inhibited the most upon CD147 knockdown in this cell line (Figure 1C).

**Figure 9:**
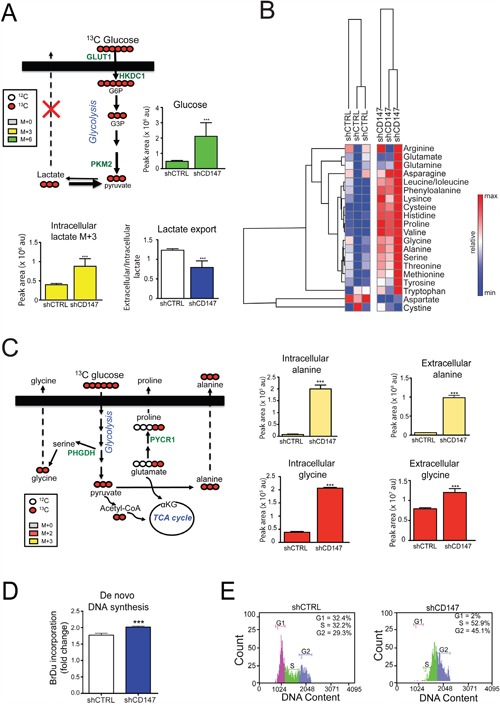
Cell growth inhibition via CD147 knockdown is linked to metabolic reprograming and cell cycle arrest **A.** and **C.** CD147 depletion leads to decreased lactate export (A) and increased production of amino acids (C). Metabolite labeling experiments with ^13^C_6_-glucose in PANC1 shCTRL and shCD147 cells. See Methods for experimental details. Proteins detected in SILAC experiments are in green. αKG - α-ketoglutarate, TCA cycle -tricarboxylic acid cycle, GLUT1 - glucose transporter 1, HKDC1 - hexokinase containing domain 1, PKM2 - pyruvate kinase isoform 2, PYCR1 - Pyrroline-5-Carboxylate Reductase 1, PHGDH - Phosphoglycerate Dehydrogenase. Bars are ± SEM, n=3, *p<0.05, **p<0.01 ***p<0.001. **B.** Neutral amino acids are specifically upregulated in PANC1 CD147 depleted cells. Heat maps summarizing the relative abundance of amino acids between the different cell lines. Relative peak intensities were clustered using GENE-E software. **D.** PANC1 cells increase their nucleotide production upon the loss of CD147 expression. 5-bromo-2'-deoxyuridine (BrDu) incorporation was monitored using ELISA assay. Bars are ± SEM, ***p < 0.001. **E.** CD147 deficient PANC1 cells are arrested in S/G2 phase. FACS cell cycle analysis.

The glucose transporter GLUT1 is highly expressed in glycolytic tumors, such as KRAS mutated pancreatic cancers, yet our SILAC experiments show that it is further upregulated upon CD147 knockdown (Figure 3A). We used metabolic tracing with glucose to determine whether GLUT1 up-regulation in CD147 depleted cells results in increased glucose uptake. Indeed, elevated levels of labeled glucose (M+6) were observed in CD147 depleted cells (Figure 9A), followed by a significant decrease in lactate export (Figure 9A). Since inefficient lactate removal promotes a negative inhibitory feedback on early rate limiting enzymes of glycolysis such as phosphofructokinase, increases in labeled glucose in CD147 depleted cells can be explained either by a metabolic bottleneck in early glycolysis or by compensatory mechanisms that promote an up-regulation of GLUT1 to increase glucose uptake in attempts to accommodate for impaired glycolysis. Through mass action law, a bottleneck in late glycolysis would promote the build-up of lactate precursors, which could in turn fuel anabolic reactions using the excess carbon backbones to produce amino acids through transamination. Since a primary amine group source for transamination in cells is glutamine-derived glutamate, we hypothesized that glutaminolysis should be enhanced in CD147 depleted cells. Indeed, an increased utilization of glutamine was also confirmed in CD147 depleted cells cultured in the presence of isotopically labeled glutamine (^13^C_6_,^15^N_2_-glutamine) (Supplementary Figure 9A). This was also in agreement with our findings of elevated expression of ASCT2 (Supplementary Figure 4B), a glutamine and neutral amino acid transporter and further implies that CD147 depleted cells rely on glutamine as an amine group donor for fueling anabolic reactions and carbon backbone donor to replenish the reservoirs of TCA cycle intermediates.

Our SILAC and cross-linking/MS analysis revealed deregulation of cellular transporters and enzymes involved in amino acid synthesis with CD147 depletion (Figure 3A), which was consistent with the increased biosynthesis of several neutral amino acids (Figure 9B). The use of a glucose and glutamine tracers revealed significant rises in intracellular labeled alanine (M+3), proline (M+2 and M+5+1), and glycine (M+2), and extracellular alanine (M+3 and M+0+1), proline (M+5+1), and glycine (M+0+1) in CD147 depleted cells (Figure 9C and Supplementary Figure 8B). Based on our flux data that proline production can be fueled by glutamine-derived glutamate, monitoring proline labeling is an appropriate readout of glutamine utilization. Our results show significant labeling in proline from heavy glutamine, which corroborates the above data of increased glutamine utilization (Supplementary Figure 8B). Furthermore, excess lactate could allosterically inhibit the forward reaction of lactate dehydrogenase, thereby resulting in the accumulation of pyruvate that would then act as a substrate for alanine production by way of glutamine-fueled transamination. Our steady state and flux metabolomics analysis demonstrated elevated alanine production/export in CD147 knockdown cells (Figure 9B–9C), thus representing an adaptation mechanism to account for increased intracellular lactate that is further supported by increased levels of amino acid transporters (Supplementary Figure 4B). Interestingly, significant increases in labeled glycine were observed in the media of ^13^C ^15^N-gluatmine incubated CD147 knockdown cells (Supplementary Figure 8B), consistent with the hypothesis that CD147 depleted cells fuel anabolic pathways to detoxify excess carbon backbones derived from the glycolytic bottleneck upon deregulated lactate export. This is particularly important since we also detected increased levels of PHGDH, an enzyme that catalyzes a rate-limiting step in serine biosynthesis (Figure 3A). Serine biosynthesis is a prerequisite for glycine generation and subsequent nucleotide synthesis. Indeed, BrdU incorporation assay indicated slight increase in *de novo* DNA synthesis (Figure 9D and Supplementary Figure 8C–8D) pointing to a possibility of increased nucleotide synthesis in CD147 depleted cells.

The increased *de novo* DNA synthesis and decrease in cell proliferation could be the primary link between CD147 metabolic reprogramming and the observed phenotypes. Therefore, we next analyzed the cell cycle profile in CD147 knockdown cells and observed cell cycle arrest in S/G2 phase (Figure 9E and Supplementary Figure 8E). Such cell cycle arrest supports the supposition that CD147 knockdown cells remove glycolytic intermediates by producing building blocks for cell growth. Furthermore, the accumulation of metabolic intermediates can lead to an increase in cellular toxicity and cell death and, hence, we next measured cell death via Annexin V staining. We did not observe a significant change in cell viability due to the loss of CD147 (Supplementary Figure 8F), which further supports the cycle arrest as a primary mean of inhibiting cell growth upon CD147 depletion. Taken together our metabolic analysis reveals that CD147 loss results in metabolic reprogramming and activation of compensatory pathways to counteract the detrimental effects of CD147 depletion.

### Silencing of CD147 decreases tumor growth and xenograft tumor metabolism *in vivo*

**Figure 10:**
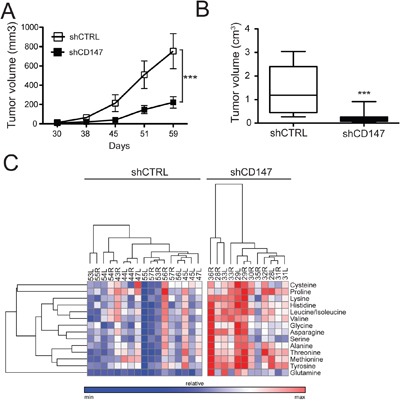
CD147 drives tumor growth in a xenograft mouse model and supports CD147-mediated metabolic reprogramming **A.** Tumor growth is significantly diminished in the absence of CD147. Growth curves of control or shCD147 (#1 and #3) PANC1 xenografts in nude mice. Equal amounts of shCTRL or shCD147 (#1 and #3) cells were injected into null mice. Mice were aged until tumors reached ~2,500 mm^3^ or all control tumors reached that size. ***p < 0.001. **B.** Decreased tumor volume is consistent with CD147 downregulation. Equal amounts of shCTRL or shCD147 (#1 and #3) cells were injected into null mice. Mice were aged until tumors reached ~2,500 mm^3^ or all control tumors reached that size. Tumor volumes were reported on the day of animal sacrifice. Bars are ± SEM, n=15, ***p<0.001. **C.** Amino acid levels are significantly augmented in CD147 depleted tumor tissues. Heat maps summarizing the relative abundance between different tumors for the detected amino acids. Relative peak intensities were clustered using GENE-E software.

To correlate the *in vitro* consequences of CD147 silencing with an *in vivo* system, we injected PANC1 CD147 depleted (shCD147#1 and shCD147#3) or control cells subcutaneously into the left and right flank of nude mice. Animals were sacrificed and tumors excised when all control tumors reached 2-2.5 cm^3^. Consistent with our cell culture analysis, CD147 depletion led to a significant attenuation of PDAC tumor growth and tumor size (Figure 10A–10B). Although shCD147#3 lost CD147 silencing over time, both in cell culture (Supplementary Figure 1A) and in some of the mouse xenografts (Supplementary Figure 9A), low tumor size and diminished tumorigenic growth for the tumors that retained CD147 depletion were observed (Figure 10B). Though our cell culture analysis showed increased intracellular lactate levels in CD147 knockdown cells (Figure 2D), we were unable to directly assess the levels of intracellular versus secreted lactate in homogenized tumor tissue. On the other hand, no significant differences in in tumor lactate levels were observed between control and CD147 depleted tumors (Supplementary Figure 9B), suggesting that the beneficial effects on tumor size diminishment in CD147-silenced xenografts, may be marginally dependent on the role of CD147 as an ancillary protein for MCTs. This is likely due to the fact that homogenized tumor tissue represents a mixed population of cancer cells and the stromal environment and our analysis reflects total metabolite levels rather than cell or stromal specific populations. Alternatively, fueling of anabolic reactions using glycolysis-derived carbon backbones would result in the promotion of anabolic reactions that *in vitro* are limited by the closed system (equilibrium between intracellular/media contents), while *in vivo* these reactions are less constrained by a continuous clearance of circulating amino acids by specific organs, such as the liver, or simply by excretion (e.g. urine). In agreement with this hypothesis, and in accordance with the *in vitro* analysis, CD147 knockdown tumors indeed showed increased levels of amino acids (Figure 10C and Supplementary Figure 9C).

Finally, the observation in our cell culture model that CD147 downregulation leads to decreased expression of proteins important for stromal and extracellular matrix remodeling (ECM) such as collagen A1 and TGM2 (Figure 3A and Supplementary Figure 3B) persisted in tumor tissue samples where another ECM component, fibronectin (FN), was also downregulated in CD147 silenced tumors (Supplementary Figure 9D). These data are also in agreement with the MET detected in CD147 silenced cells, since increased expression of FN is directly correlated to mesenchymal phenotypes [31]. Taken together, our *in vivo* analysis is consistent with our *in vitro* studies, where we have identified the functional consequences of disrupting CD147 interactions.

## DISCUSSION

Our analysis reveals that CD147 interacts with multiple proteins and acts as an ancillary protein for these interacting partners via its ability to protect against degradation and assist in proper membrane translocation. CD147 has previously been described to play a chaperone role for MCT1 and MCT4; however, our study identifies other CD147 interacting patterns beyond MCTs, which include PMCA1. Molecular chaperones typically function in large complexes with other co-chaperone proteins. For example, heat shock proteins (HSPs) form multi-subunit structures, where different accessory proteins play diverse functions, and thus, chaperone interaction networks are often composed of many proteins [32]. Consistent with these phenomena, our cross-linking/pull-down analysis identified a multitude of CD147 interactions, including known chaperones and secretory proteins (i.e., calnexin, calreticulin, Syntaxin 4, CD98hc). For example, CD98hc is an ancillary protein for several neutral amino acid transporters and the presence of CD98hc in CD147 cross-linking/pull-down samples, without a parallel inhibition of CD98hc levels in CD147 silenced cells, may suggest a co-chaperone function in regard to its interaction with CD147. Our data suggest that CD147 is a chaperone for several proteins that include MCTs, as well as other transmembrane proteins.

A clear functional role of CD147 function in PDAC cells is its interaction with multiple proteins involved in small molecule and ion homeostasis such as lactate and calcium, the levels of which responded significantly to CD147 depletion. Although we established that cellular localization of MCTs is reliant on CD147 expression in PDAC, their independent downregulation leads to different phenotypic changes that prompted us to thoroughly identify and characterize additional CD147-protein interactions. To this end, we identified another important CD147 interacting partner in PMCA1, which is a plasma membrane ATPase responsible for maintaining intercellular calcium balance and extracellular calcium efflux [25]. Increased intracellular calcium is implicated in the regulation of cell cycle specifically via the activity of calcium binding calmodulin-kinase II, which influences S and G2/M progression. Interestingly, our data show that CD147 depleted cells are arrested in S/G2 phase [33]. Taken together, the inhibition of CD147 interactions leads to alterations in calcium homeostasis and global changes to the metabolome and proteome that synergistically affect vital cellular processes such cell cycle arrest and consequent cell growth.

Although CD147 has previously been proposed to play a chaperone role for MCTs, the molecular details of CD147 interactions and potentially other functions have not been fully portrayed. Here, we specifically tested the contribution of various mutations in CD147 to its interactions and translocation mechanism, ultimately showing that the TM region plays a critical role in maintaining CD147 interactions. This was exemplified by substituting CD147 TM residues with TM regions derived from other single pass transmembrane proteins to show that although these chimeras were capable of translocating to the cellular membrane, the interactions with other proteins were lost. The roles of TM regions in protein-protein interactions have been largely understudied, mainly due to difficulties with assessing such interactions through biophysical studies. However, recent studies on Toll-like receptors (TLRs) have underscored the importance of homo- and hetero- interactions for these single pass transmembrane proteins activity [34], as we have shown here for CD147 mediated cellular membrane localization of MCT1, MCT4 and PMCA1. Furthermore, Manoharan et al. previously revealed that the charged residue (E218) within the CD147 TM region plays a role in MCTs engagement and thus, these data are consistent with our findings here [35]. Our analysis provides evidence that CD147 interactions are mediated by the TM region and link these TM-specific interactions to the phenotypic changes mediated by CD147.

The development of therapeutic strategies entails a comprehensive understanding of molecular mechanisms; therefore, we aimed to acquire a detailed analysis of CD147 functions in PDAC. CD147 depletion led to augmented glucose and glutamine uptake and altered metabolic flux, supported by varied levels of metabolic proteins (i.e., GLUT1, HKDC1, PKM2, ASCT2). For example, HKDC1 has been previously shown to correlate with KRAS signaling in cancer cells, where loss of mutated KRAS expression led to an increased HKDC1 expression [36]. Increased HKDC1 levels were further explained by a formation of a negative feedback loop to maintain high levels of glycolysis in the absence of KRAS signaling, a common feature of cancer cells [37]. Thus, the loss of CD147 can lead to a similar negative feedback loop in which HKDC1 and GLUT1 upregulation compensate for the metabolic reprogramming of CD147 depleted cells, which is consistent with increased abundance of many metabolites (i.e., lactate, amino acids) and proteome changes (i.e., GLUT1, ASCT2, LAT1). Furthermore, our observations of decreases in PKM2, an essential enzyme important for the regulation of glycolysis, is consistent with previous studies showing inhibition of cell growth and metabolic reprogramming in PKM2 silenced PDAC cells [38]. Lastly, the expression and cellular localization of GLUT1 and LAT1 has previously been linked to CD98hc expression, and thus our detection of upregulated CD98hc expression along with increased expression of its interacting partners further supports the existence of a feedback loop [39]. Taken together, this unique adaptation to intracellular lactate accumulation due to CD147 depletion is an efficient mechanism to protect cells from apoptotic cell death.

In summary, our data suggest that CD147 assists small molecule and ion transporters by preventing their proteasome-dependent degradation and targeting them to the cellular membrane. Through this mechanism, CD147 indirectly regulates several signaling events, including cell cycle arrest, calcium signaling, the EMT, and metabolism, which ultimately affects cell proliferation *in vitro* and *in vivo*. A large contribution to CD147 mediated function is due to its connection with MCTs, however our data suggest that other interactions are important as well. We have shown an interaction between CD147 and a subset of membrane proteins, our cross-linking/MS analysis identified several other proteins that could interact with CD147, but were not further characterized in here. Our observation that some of these proteins were upregulated due to the loss of CD147 expression suggests the formation of a large complex with CD147 rather than direct interactions. In this regard, targeting a central complex component such as CD147 could have a more drastic effect in blocking cancer progression rather than specifically targeting interacting partners. Recent discoveries using MCT pharmacological inhibitors provide evidence for a role of CD147 and MCTs in regulating cellular metabolism via its ancillary function in several cancer phenotypes [6, 14]. Our data support these previous studies and provide rationale for developing selective impasse strategies to inhibit CD147 activity in PDAC progression, either alone or in combination with pharmacological inhibitory treatments of amino acid anabolic reactions or MCTs.

## MATERIALS AND METHODS

### Cell lines and culture conditions

Human immortalized pancreatic cells (NT1, NT2, NT5) were described previously [40]. L3.6pl and L3.5 cells were a kind gift from Dr. Isaiah J. Fidler, Department of Cancer Biology, University of Texas MD Anderson Cancer Center [41]. Pancreatic adenocarcinoma cell lines HS-766T, BxPC3, PANC1, MiaPaCa2, were purchased from ATCC. All cells were cultured in DMEM media supplemented with 10% fetal bovine serum (FBS), 0.1g/ml penicilin/streptomyocin. FreeStyle 293-F cells (Thermo Fisher) were cultured in FreeStyle 293 Expression Medium (Thermo Fisher). All cells were routinely tested for mycoplasma contamination.

### Reagents and plasmids

MCT1 inhibitor, ARC-155858 (TOCRIS, UK), MG132 (Fisher Scientific) and DSS (dissucinimidyl suberate, Thermo Fisher) were dissolved in DMSO and used at the indicated concentrations. For each compound dissolved in DMSO the corresponding concentration of DMSO was used as a control never exceeding 0.5% DMSO. Sulfo-NHS-SS-Biotin was purchased from Thermo Fisher. E-cadherin, β-catenin, EGFR, ICAM1, CD98hc, LAT1, ITGβ4, PKM2, G6PD, HKDC1, β-actin antibodies were all from Cell Signaling; CD147 (sc-21746 and sc-13976), MCT4 (sc-50329 and sc-376139), MCT1, ASCT2 were from Santa Cruz Biotechnology; GLUT1 was from Abcam and Fibronectin from R&D Systems. All fluorescently conjugated Alexa antibodies were purchased from Invitrogen. All plasmids were purchased from Functional Genomics Facility at the University of Colorado Cancer Center Shared Resource and included: TRCN0000006732 (shCD147 #1), TRCN0000006733 (shCD147 #2), TRCN0000006734 (shCD147 #3), TRCN0000038474 (shMCT4 #1) and TRCN0000038476 (shCD147 #2).

pLenti CMV/TO Neo empty (w215-1) was a gift from Eric Campeau (Addgene plasmid # 17485). CD147-GFP plasmid was cloned in house into pcDNA3.1-GFP with an N-terminal signal peptide and a C-terminal GFP. Point mutations and deletion constructs were generated with Quick Change Site directed mutagenesis kit (Agilent Technologies, Santa Clara, CA) according to manufacturer's description. For CD147-ECD construct residues corresponding to a signal peptide (amino acids 1-21 of human CD147) followed by FLAG tag, 6xHis affinity tag, a TEV cleavage site, and amino acids 22-2-5 of human CD147, were cloned into a mammalian expression vector (pCEP4, Invitrogen) between NdeI and BamH1 sites using In Fusion cloning strategy (Clontech). pMM2-hPMCA1b was a gift from John Penniston & Emanuel Strehler (Addgene plasmid #47758). To generate pMM2-hPMCA1b-GFP plasmid, GFP was PCR amplified from pcDNA3.1-GFP plasmid and inserted using KpnI site (In-Fusion Cloning, Clontech). Cyclophin-A was generated as previously described ^37^. CD98hc ORF and Syndecan-1 ORF were purchased from Functional Genomics Facility, UCD Cancer Center. The extracellular region of CD98hc (aa 212-630) and the extracellular region of Syndecan-1 (aa 23-251), respectively were cloned in house into pet15b containing an N-terminal 6xHis and thrombin cleave site. 6xHis tagged GFP-nanobody was synthesized into pJ401 plasmid (DNA2.0, Menlo Park, CA).

### Stable cell lines generations

Stable cell lines were generated as previously described [42]. Knockdown was confirmed by immunoblotting analysis and qRT-PCR, indicating approximately 70% - 90% depletion. Similarly, lentiviral particles encoding human BSG ORF in pLenti CMV/TO vector or empty vector were transduced into knockdown cells using standard protocols. For different CD147-GFP or GFP control stable cells, shCTRL or shCD147#1 cells were transfected using Lipofectamine 300 (Invitrogen) as per manufacturer protocol. Stable incorporation was confirmed by immunoblotting analysis, qRT-PCR or flow cytometry analysis.

### Recombinant protein purification

CD147-ECD was expressed as previously described [43]. CD98hc-ECD, Syndecan-1 and GFP-nanobody were all expressed in E. Coli strain BL21 (DE3) and purified as previously described [29]. Briefly, cell pellets were lysed via sonication in lysis buffer (50 mM Na_2_HPO_4_, pH 7.5, 500 mM NaCl, 10 mM imidazole), applied to a 10-20 ml Ni-sepharose column, and eluted with nickel elution buffer (50 mM Na_2_HPO_4_, pH 7.5, 500 mM NaCl, 400 mM imidazole). Elutions comprising 6xHis-tagged proteins were further purified using gel filtration into their respective buffers. CD98hc and Syndecan-1 were concentrated to approximately 0.5-1.5 mM with 95% H_2_O/5% D_2_O for subsequent NMR analysis. Purified GFP-nanobody was conjugated to NHS-activated Sepharose Fast Flow resin (GE Healthcare, Pittsburgh, CA) according to manufactures protocol.

To express glycoylated CD147-ECD mammalian expression system was utilized. Transient transfections of FreeStyle 293-F cells (Invitrogen) with PEI (Polyethylenimine, Fisher Scientific) were performed as previously described with slight modifications [44]. Briefly, the day before transfection cells were passaged into fresh media to final concentration of 1x10^6 cells/ml and allowed to grow overnight. On the day of transfection cells were spun down and re-suspended to 2.5-3x10^6 cells/ml in fresh medium supplemented with 3ug/ml of DNA and 9ug/ml of PEI, and allowed to grow overnight. Next day, cells were diluted to 1x10^6 cells/ml with fresh medium supplemented with VPA (Valproic acid, Sigma) at a final concentration of 2.2 mM. Cells were then allowed to grow for 5 days and conditioned media was collected on day 5.

Secreted protein was purified from culture media after removal of cells via centrifugation for 20min at 8000rpm and culture media filtration through 0.22μm. Typically, 1L of total culture media was purified at a time. Immediately before IMAC chromatography 0.2L of 5X Ni buffer A1 (250 mM phosphate, 2.5 M NaCl, 50 mM Imidazole, pH 7.5) was added to the media and the whole volume was applied to pre-equilibrated 10ml Ni-high affinity column using AKTA FPLC system (GE Healthcare). 6xHis tagged protein was eluted with increasing concentration of Ni buffer A1 + 400mM Imidazole and concentrated to about 1ml for subsequent gel filtration chromatography (S75 column). Gel filtration was performed in final buffer containing 50 mM phosphate 50 mM NaCl pH 6.5 for NMR or 1X PBS for cell culture treatments. Protein purity was evaluated by SDS-page gel electrophoresis and measurement of OD ratio at 260/280.

### NMR spectroscopy

All NMR spectra were collected at 25 °C on a Varian 600 MHz or 900-MHz spectrometer with samples supplemented with 5% D_2_O. All spectra were processed using NMRPipe software [45] and analyzed using CCPNmr software [46]. Unless otherwise noted, all pulse sequences were obtained from standard Varian Biopack libraries.

### Immunoblotting, cross-linking, cell surface biotinylation and pull-downs

Cells were grown in 10 cm dishes and on the day of experiments incubated with 0.5 mM DSS or PBS for 30 min in RT. Cross-linking was quenched by addition of 1M Tris, cells washed with 3 times with 1X PBS and lysed in RIPA buffer. For cell surface biotinylation 10-15x10^6^ cells were washed twice with ice cold PBS and re-suspended in 1 ml of 1X PBS pH 8. Sulfo-NHS-SS-Biotin was added to a final concentration of 0.4 mM and cells were incubated in RT for 30 mins following three washes with 1X PBS and cell lysis in RIPA buffer.

Equal amount of total cells lysate (500 μg) was applied to 40 ul of GFP-nanobody conjugated beads or strepavidin magnetic beads (NEB) and incubated on a rotating shaker for 1 hr in 4 °C. Beads were then washed four times with 500 ul of RIPA buffer and 40 ul of 1X SDS loading buffer, containing reducing agents was added to the beads, heated to 100 °C for 10 min and resolved on NuPAGE Bis-Tris 4−12% gradient gel (Invitrogen). Gels were stained with Coomassie for Proteomics analysis (see Proteomics analysis) or transferred to PVDF membranes and processed for immunoblotting as previously described [47].

### Immunofluorescence and proximity ligation assay (pla) analyses

Cells were grown in 8-well chamber slides and following treatment or transfections, when applicable, cells were fixed in either cold 100% methanol for 8mins in −20°C or 2% paraformaldeyde (for GFP signal evaluation) in 1X PBS for 15mins in RT. Cells were then washed with 1X PBS, and blocked with Knudsen Buffer (1x PBS, 0.5%BSA, 0.5% NP-40, 1mM MgCl2, 1mM NaN3). Cells were stained with the specific primary antibodies followed by appropriate secondary antibodies incubations. DNA was visualized using Hoechst 33342 (Sigma). Coverslips were mounted using Citifluor AF-1 (Ted Pella) mounting media.

Protein interactions were analyzed *in situ*, using Duolink II PLA detection kit (Sigma Aldrich, St. Louis, MO) as per manufacturer's instructions. Briefly, cells were processed same as for immunofluorescence, but Duolink specific secondary antibodies were utilized instead, followed by probe ligation and amplification. The specificity of each interaction was tested by omitting one of the primary antibodies in the assay preparation. All images were visualized with a Nikon Ti Eclipse inverted microscope (Nikon, Melville, NY) with a Nikon 100x PlanApo numerical aperture 1.4 objective. Images were captured with and Android iXon electron-multiplying charge-coupled device (CCD) 888E camera (Andor Technologies, Belfast, United Kingdom). Image processing was performed using Image J software [48]. PLA interactions were quantified with Cell Profiler software (Cell Profiler version 2.1.1).

### Cell apoptosis measurements and cell cycle arrest analysis

Cells were grown to 80–90% confluence prior to FACS analysis. The cells were trypsinized, washed twice with FACS buffer (1X PBS containing 1% BSA and 5 mM EDTA), and re-suspended in the same buffer to a concentration of 10 x 10^5^ cells/100 μL. For cell apoptosis measurements cells were stained with Annexin-V-FITC antibody (Thermo Fisher) following flow cytometry using a Guava EasyCyte flow cytometer (EMD Millipore, Billerica, MA). FITC labeled IgG isotope control was used as a negative control. For cell cycle analysis cells were permeabilized with 2% paraformaldehyde and stained with PI. Cells were then fixed with 1% PFA/1X PBS and analyzed by flow cytometry using a Guava EasyCyte flow cytometer (EMD Millipore, Billerica, MA). Side scatter and forward scatter profiles were used to eliminate cell doublets.

### Cell growth and migration

On day 1, cells were plated into 6-well plates at 0.2 x 10^6^ cells/well in complete media and allowed to adhere for 24hrs. On day 2, and every day after for next 3-4 days, cells were trypsinized with 100 μL trypsin, re-suspended in 900 μL of complete media and seeded back into the same plate wells. Each day a 10 μL aliquot was stained with Tryptan blue and cell count was monitored using Countess cell counter (Thermo Fisher). All cell growth data was normalized to day 1. Migration was assayed using 8-μm pore Boyden chambers (Costar, Boston, MA). 200 uL of cells, at 1 x 10^6^ cells/ml was plated in the top chamber of the transwell insert and 500 uL of media containing the indicated chemoatractant (FBS) was placed in the lower chamber. Cells were allowed to migrate for 4hrs at 37°C in a humid atmosphere at 5% CO_2_. After incubations, cells were removed from top well and filters were incubated in 250 uL of 1X Cell Dissociation Solution (Trevigen, MA). Filters were discarded and dissociated cells were lysed by freeze-thawing. Finally, cell lysates were incubated with CyQuant reagent (Thermo Fisher) and signal was read at 480/520 using fluorescence plate reader.

### Calcium flux

Calcium flux analysis was performed using Fluo-4 AM calcium flux assay (Invitrogen) according to the manufacturer protocol. Briefly, on the day of analysis cells were loaded for 60 mins with 5 μM Fluo-4 dye in calcium flux buffer. Fluorescence signal was recorded using florescence plate reader (Glowmax, Promega) before (background) and after stimulation with 10 μM ionomycin. The data are normalized to the background signal.

### Enzyme-linked immunosorbent assays (ELISAs)

ELISA assays with glyCD147-ECD were performed as previously described [49]. Measurements were carried out as per manufacturer's protocol (ELISA Tech, Aurora, CO). For BrdU (5-bromo-2'-deoxyuridine) incorporation cells were plated in 96-well plates and allowed to adhere for 24 hrs. Next day BrdU reagent was added to each well and cells were analyzed for BrdU incorporation 48 hrs later using BrdU cell proliferation assay (Cell Signaling) according to the manufacturer instructions.

### Inhibitor treatments

Cells were grown to 40–60% confluence prior to treatments. The cells were treated with indicated concentrations of ARC-155858 (ARC) in full serum media for 72 hrs at 37°C in a humid atmosphere at 5% CO_2_. For proteasomal inhibition cells were treated with indicated concentrations of MG132 in full serum media for 16 hrs at 37°C. Following treatment cells were processed for metabolomics, microscopy or lysed for WBs.

### Animal studies

For the *in vivo* experiment, 5-6 week old female athymic nude mice were obtained from Envigo. 1 million PANC1 (shCTRL or shCD147#1, shCD147#3) cells were subcutaneously injected in a 1:1 ratio of DMEM media and matrigel (Fisher Scientific) into the right and left flanks of mice, such that each mouse received injections of the same cell type. Tumor volume was measured weekly and the animals were sacrificed when tumors reached 2-2.5cm^3^ (volume = 0.4 × width^2^ × length). Tumors were further processed for immunoblotting after protein extraction in RIPA buffer or metabolomic analysis as described in the metabolomics section.

### Proteomics analysis

For SILAC analysis DMEM was supplemented with dialyzed serum and heavy - Arg-^13^C/Lys-^13^C, or light - Arg-^12^C/Lys-^12^C (ThermoScientific). shCD147 cells were cultured in the heavy media and shCTRL cells were cultured in the light media. Cells were allowed 8 doublings before 98% isotope incorporation was achieved. The incorporation was tested via MS and for final analysis cells were mixed 1:1 based on the total protein content. Samples were resolved on a 1.5 mm thick NuPAGE Bis-Tris 4−12% gradient gel (Invitrogen). After excision, gel pieces were prepared for MS analysis as previously described [50].

Samples were analyzed on an LTQ Orbitrap Velos mass spectrometer (Thermo Fisher Scientific) coupled to an Eksigent nanoLC-2D system through a nanoelectrospray LC − MS interface for SILAC or Q Exactive mass spectrometer (Thermo Fisher Scientific) coupled om-line to nanoflow HPLC instrument (Easy nLC 1000 UHPLC, Thermo Fisher Scientific) through a nanoelectrospray ion source (Proxeon) for cross-linked cells. Data acquisition was performed using the instrument supplied Xcalibur™ (version 2.1) software. The mass spectrometers were operated in the positive ion mode. MS SILAC data were processed using the MaxQuant computational platform and searched with Andromeda search engine against the human UniProt database.

Q Exactive MS/MS spectra were extracted from raw data files and processed for further analysis as previously described [50]. Scaffold (version 4.3.2, Proteome Software, Portland, OR, USA) was used to validate MS/MS based peptide and protein identifications. Peptide identifications were accepted if they could be established at greater than 95.0% probability as specified by the Peptide Prophet algorithm. Protein identifications were accepted if they could be established at greater than 99.0% probability and contained at least two identified unique peptides. Gene ontology analysis was performed using the DAVID Bioinformatics Database (DAVID Bioinformatics Resources, http://david.abcc.ncifcrf.gov/) with the human gene name identifiers of proteins identified to be significantly (p<0.05, protein identified in at least two out of three biological replicates) upregulated or downregulated SILAC analysis. Calculation of over-represented GO terms was performed using the entire list of identified proteins as background (threshold count = 2; EASE score = 0.1). Terms with a p-value <0.05 were selected, log10-transformed and hierarchically clustered using GENE software.

### Metabolic tracing experiments

Equal number of cells was plated into 6-well plates and allowed to adhere for 24-48 hrs. Cells where then washed with 1X PBS and media replaced with media containing 10% dialyzed serum and the indicated isotopically enriched carbon sources (^13^C_6_-glucose or ^13^C_6,_^15^N_2_-glutamine). The control experiment was performed alongside with cells exposed to media containing 10% dialyzed serum and ^12^C_6_-glucose and ^12^C_6,_^14^N_2_- glutamine. Glucose experiments were performed for 1 hr and glutamine for 24 hrs. After treatment, samples were processed same as for the global metabolomic analysis. 20 μL of conditioned media was also used for the analysis.

### Metabolomics analysis

Equal number of cells was plated into 6-well plates in 1 ml of complete media/well and allowed to adhere for 24 hrs. Next day cells were trypsinized, counted and metabolites were extracted with 1ml/2 x 10^6^ cells of extraction buffer (methanol:acetonitrile:water 5:3:2) immediately prior to analysis, 20 μL of conditioned media was also collected and metabolites were extracted by addition of 480 μL of extraction buffer. Samples were then agitated at in 4°C for 30 min following by centrifugation at 10,000 g for 10 min at 4°C. Protein and lipid pellets were discarded and metabolite fractions were stored at −80°C for further analysis.

Metabolomics analysis was performed as previously described [51]. Breifly, 10 μl of samples were injected into an UHPLC system (Ultimate 3000, Thermo, San Jose, CA, USA) and separated during a 3 minute isocratic gradient at 250 μl/min (mobile phase: 5% acetonitrile, 95% 18 mΩ H_2_O, 0.1% formic acid) in a Kinetex C18 column (150x1 mm i.d., 1.7 μm particle size – Phenomenex, Torrance, CA, USA). The UHPLC system was coupled online with a QExactive system (Thermo, San Jose, CA, USA), scanning in Full MS mode (2 μscans) at 70,000 resolution in the 60-900 m/z range, 4kV spray voltage, 15 sheath gas and 5 auxiliary gas, operated either in negative and positive ion mode. Metabolite assignments were performed through the software Maven^1^ (Princeton, NJ, USA), upon conversion of .raw files into .mzXML format through MassMatrix (Cleveland, OH, USA). The software allows for peak picking, feature detection and metabolite assignment using the KEGG pathway database. Assignments were further confirmed by chemical formula determination from isotopic patterns and accurate intact mass and retention times against a subset of standards including commercially available glycolytic and Krebs cycle intermediates, amino acids, glutathione homeostasis and nucleoside phosphates (SIGMA Aldrich, St. Louis, MO, USA).

### Statistical analysis

GraphPad Prism software was used for statistical analysis. Data are expressed as mean values ± SEM. Data were analyzed with Student's *t*-test between two groups or analysis of variance (ANOVA) coupled with post-hoc Bonferroni test for multiple pairwise comparisons. Probability values of *P* < 0.05 were considered to be statistically significant. For metabolomics, relative quantitation was performed by exporting integrated peak areas values into Excel (Microsoft, Redmond, CA, USA) for statistical analysis including T-Test (significance threshold for *p-values* < 0.05) and partial least square discriminant analysis (PLS-eDA), calculated through the macro MultiBase (freely available at www.NumericalDynamics.com). Hierarchical clustering analysis (HCA) was performed through the software GENE-E (Broad Institute). XY graphs were plotted through GraphPad Prism.

## SUPPLEMENTARY FIGURES AND TABLE




