# Supplementary material for: CD147: a small molecule transporter ancillary protein at the crossroad of multiple hallmarks of cancer and metabolic reprogramming
Source: Oncotarget. 2016 Dec 27;8(4):6742–62. doi: 10.18632/oncotarget.14272 (PMC5341751; doi:10.18632/oncotarget.14272)
Supplement: Supplementary file 1 [file oncotarget-08-6742-s001.pdf]

CD147: a small molecule transporter ancillary protein at the crossroad of multiple hallmarks of cancer and metabolic reprogramming

SUPPLEMENTARY FIGURES AND TABLE

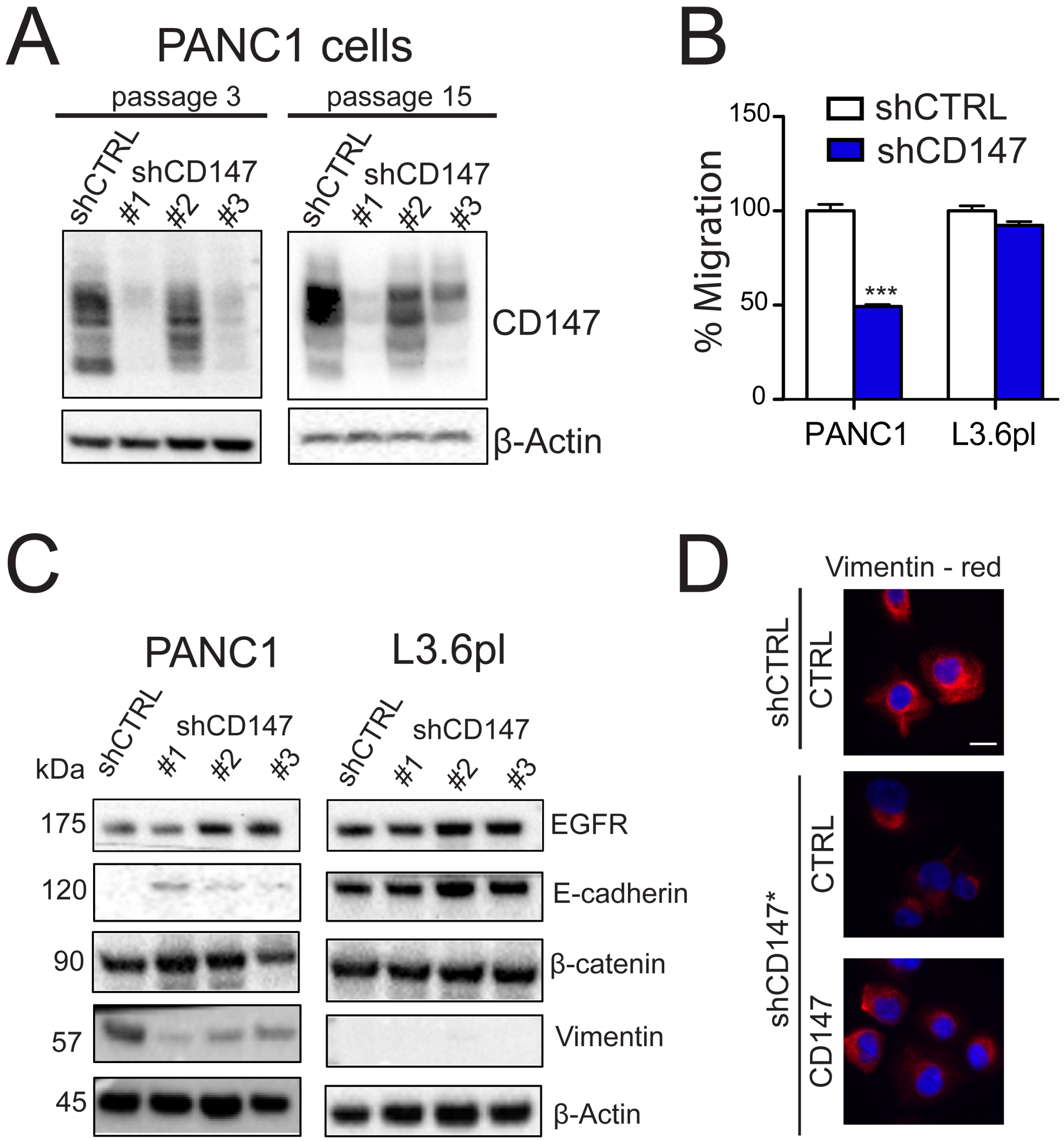

Supplementary Figure 1: The contribution of CD147 downregulation to the phenotypic changes in PDAC cell lines. **A.** CD147 downregulation does not persist over time for some of the knockdown PANC-1 cells. β-actin provided as a loading control. **B.** CD147 depletion decreases cell migration. Cell migration was assessed using Boyden chamber assay towards serum containing medium. Bars are ± SEM, ***$p < 0.001$. **C.** The level of CD147 expression affects the levels of EMT marker proteins. β-actin provided as a loading control. **D.** Rescue of CD147 expression restores the EMT. PANC1 cells were stained with Vimentin and representative images are shown. Cell nuclei were counterstained with Hoechst (blue). Scale bar, 10 µm.

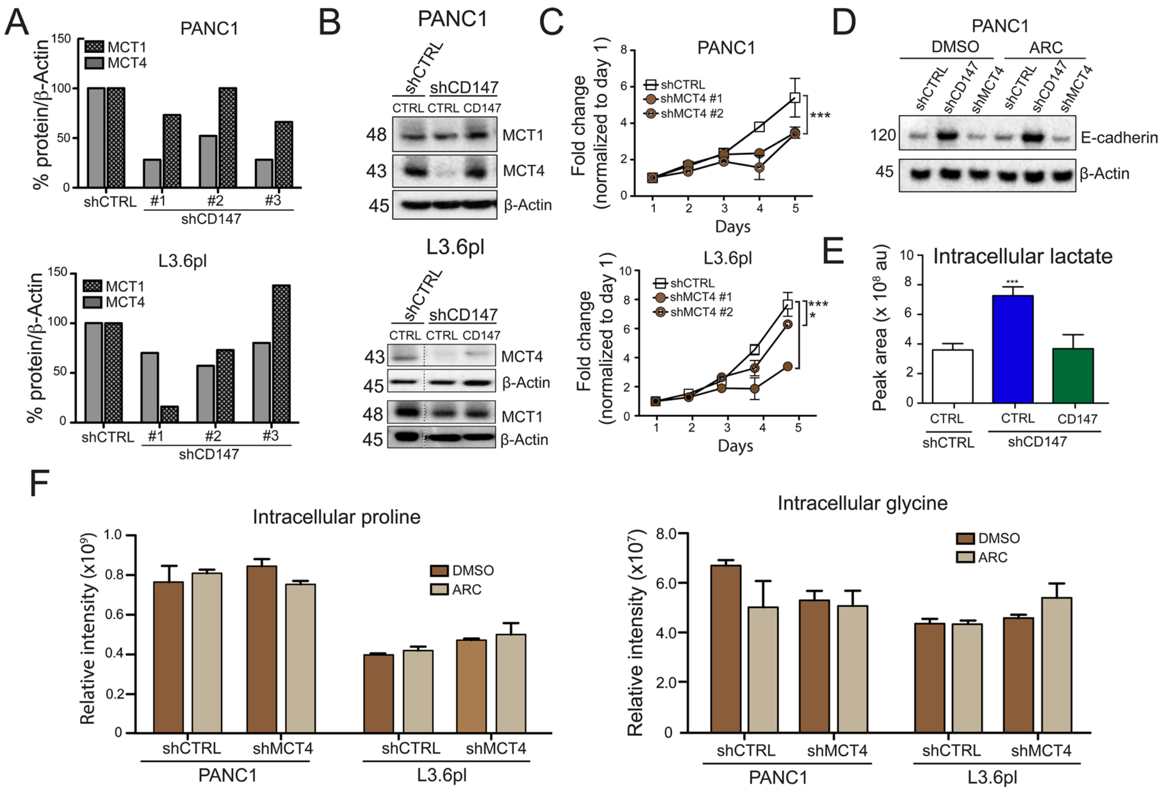

Supplementary Figure 2: CD147 function is partially dependent on MCTs. A. Densitometry analysis of Western blots from Figure 2A. Representative results are shown. **B.** Rescue of CD147 expression reinstates MCT1 and MCT4 levels. β-actin provided a loading control. L3.6pl Western is a composite of a larger gel in which all of the samples were run simultaneously. The composite was made by splicing the complete lines for presentation purposes, indicated by the dotted line. **C.** MCT4 knockdown reduces cell growth. All counts were normalized to day 1. Bars are ± SEM, n=3, ***p<0.001. Representative results are shown. **D.** The EMT is not affected when MCTs' function is inhibited. The levels of E-cadherin were measured in the PANC1 MCT4 knockdown cell line in the presence of absence of MCT1 inhibitor (ARC). β-actin provided a loading control. **E.** Rescue of CD147 expression restores intracellular lactate levels. Metabolomics analysis for intracellular lactate levels in the indicated cell lines. Bars are ± SEM, n=3, *p<0.05, ***p<0.001. **F.** CD147 knockdown cells exhibit metabolic profile distinct from MCT1 and MCT4 function inhibited cells. Metabolomics analysis for designated intracellular amino acids. Bars are ± SEM, n=3.

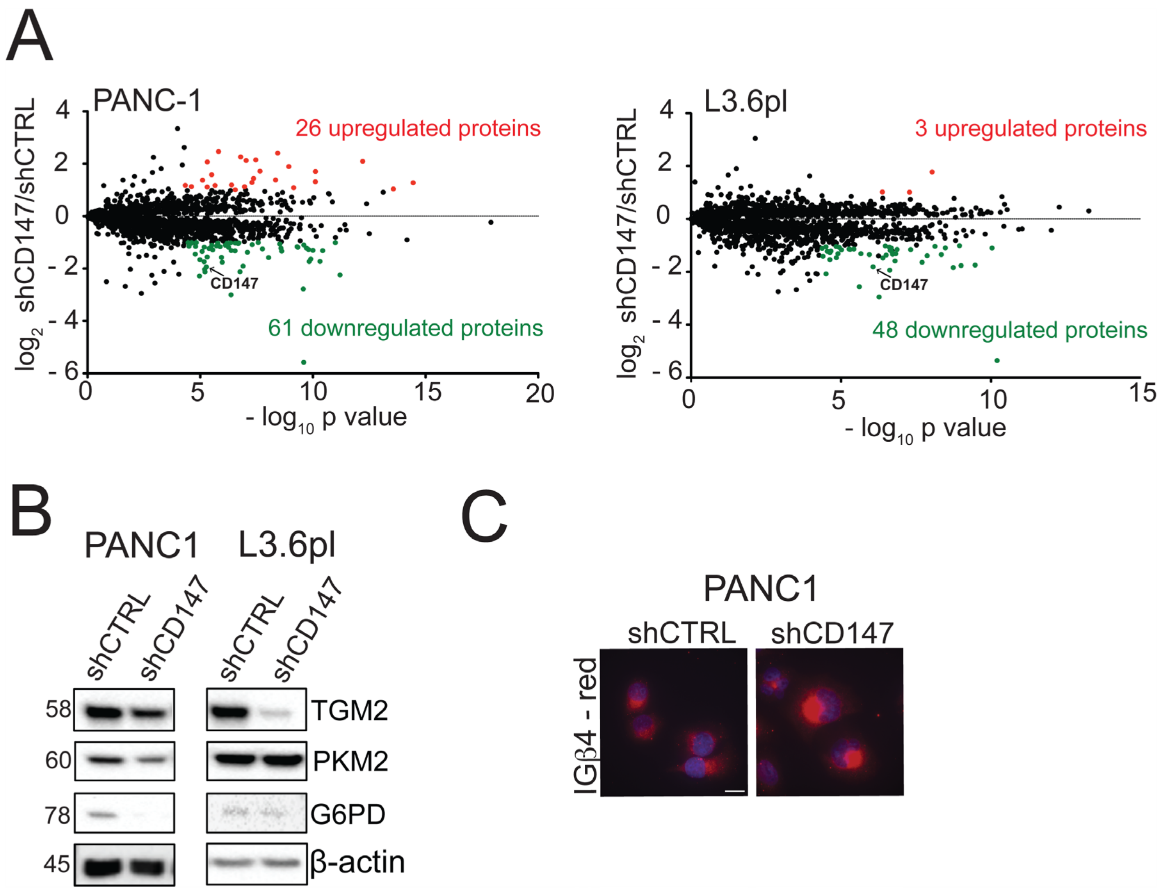

Supplementary Figure 3: Global MS analysis identified several differentially regulated metabolic and adhesion proteins. A. SILAC experiments identified a subset of differentially expressed protein in CD147 depleted cells. Volcano plots showing ratio of CD147 depleted to control cells (-log2 of shCD147/shCTRL) versus p values (-log10) of all proteins identified in 3 independent experiments from two different pancreatic cancer cell lines. Upregulated proteins are marked in red and downregulated proteins in green (p value, ANOVA). **B.** Proteomic changes identified via SILAC were validated via immunoblotting. β-actin provided a loading control. **C.** Proteomic changes identified via SILAC were validated via immunofluorescence. Cell nuclei were counterstained with Hoechst. Scale bar 10μm.

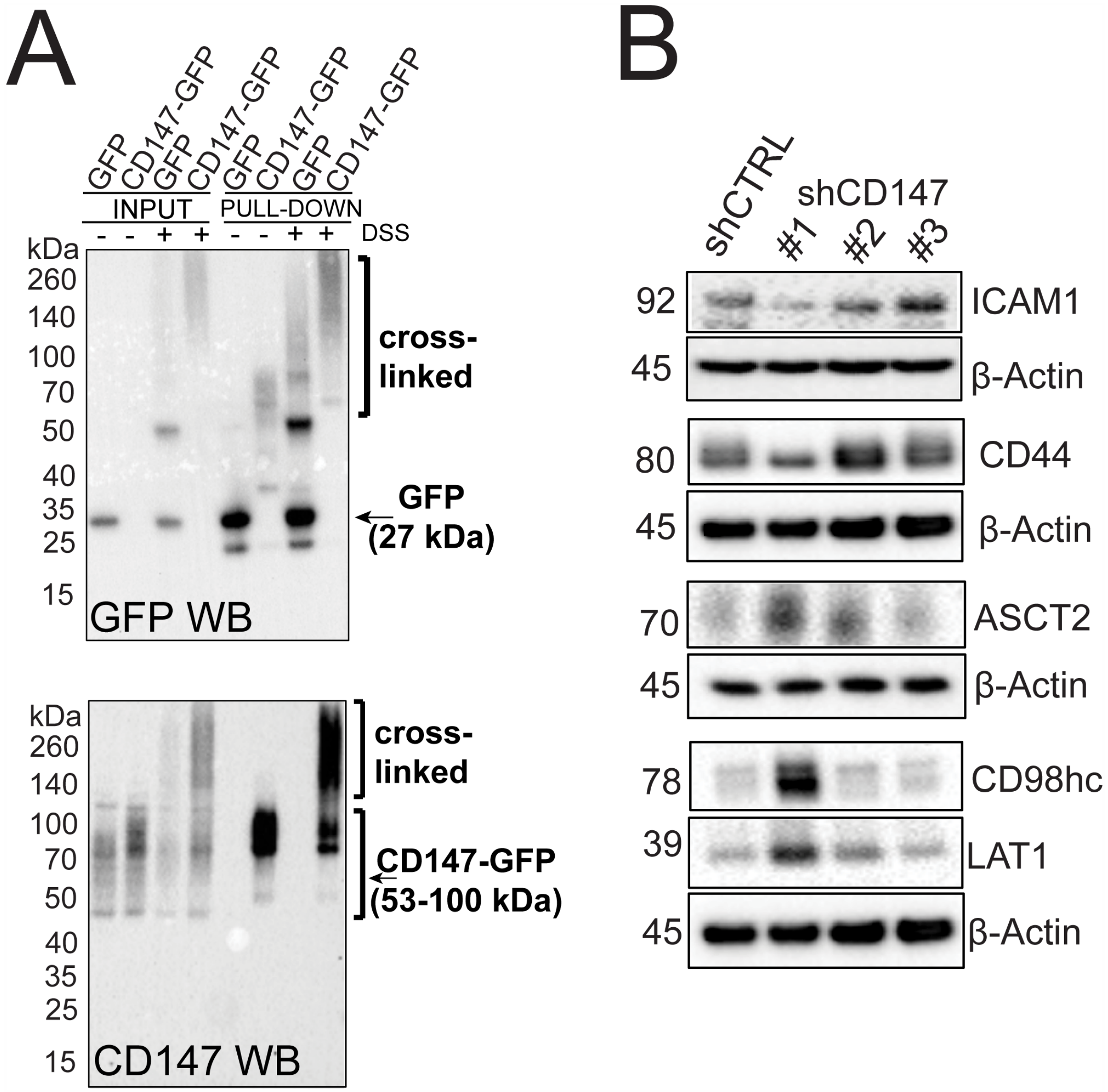

Supplementary Figure 4: CD147 genetic disruption leads to deregulation of its possible interacting partners. A. Validation of the cross-linking/pull-down approach for both GFP and CD147-GFP. Western blots conformation of the specificity of pull-downs. Samples were prepared as described in methods and the ability to pull down CD147 was confirmed via immunoblotting. Input lines provided loading controls. **B.** CD147 differentially regulates the levels of some of its possible interacting partners. β-actin provided a loading control.

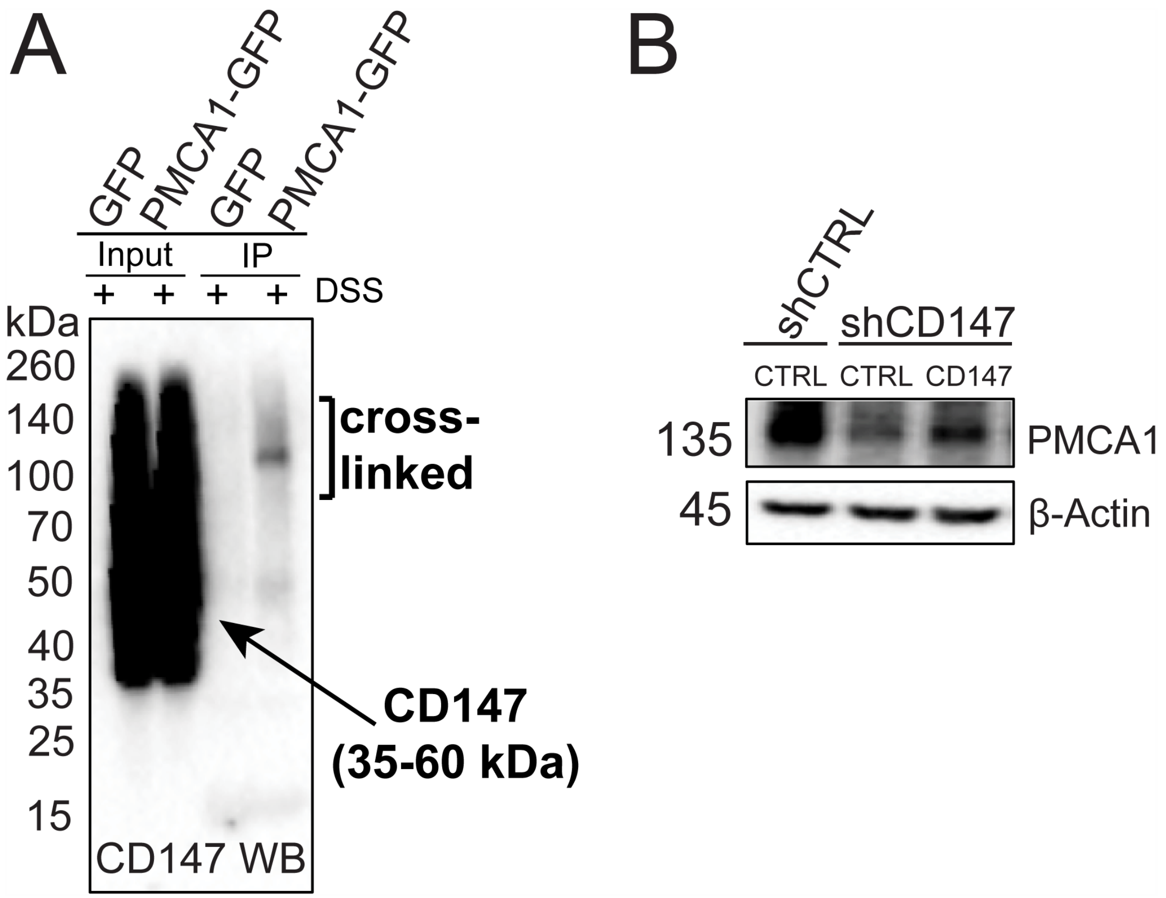

Supplementary Figure 5: Re-expression of CD147 rescues PMCA1 expression in PANC1 cells. A. Higher exposure image from Figure 6A. Pull-down of transfected PMCA1-GFP shows an association with CD147. CD147 immunoblots for control GFP or PMCA1-GFP transfections followed by DSS cross-linking in PANC1 cells. Arrows indicate the location of the correct molecular weight band for each identified proteins. Cross-linked fractions are also marked. **B.** CD147 rescue restores PMCA1 expression. β-actin provided as a loading control.

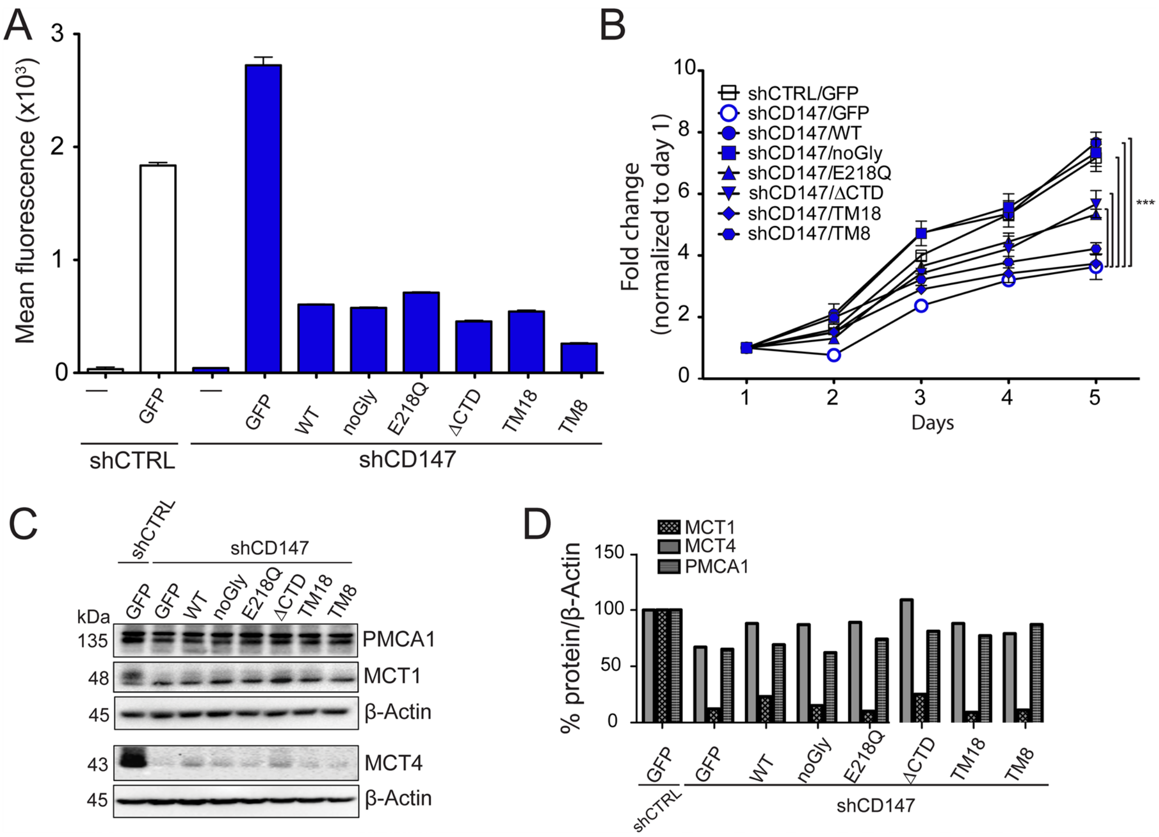

Supplementary Figure 6: The CD147 transmembrane region is essential for proper cellular localization of its interacting partners. A. The re-expression of wild type (WT) and different CD147-GFP mutants are confirmed by the analysis of GFP expression. FACS analysis of GFP control and GFP fusion cell lines represents the level of CD147 re-expression in those cells. **B.** Cell growth is affected differently depending on the different CD147-GFP constructs. All cell counts were normalized to day 1 for the indicated cell line and reported as fold changes (*p < 0.05, **p < 0.01, ***p < 0.001) **C.** CD147 re-expression leads to the restoration of its partner proteins expression. CD147-GFP constructs differentially rescue CD147 target protein expression. β-actin provided a loading control. **D.** Densitometry analysis of Western blot from C. Representative results are shown.

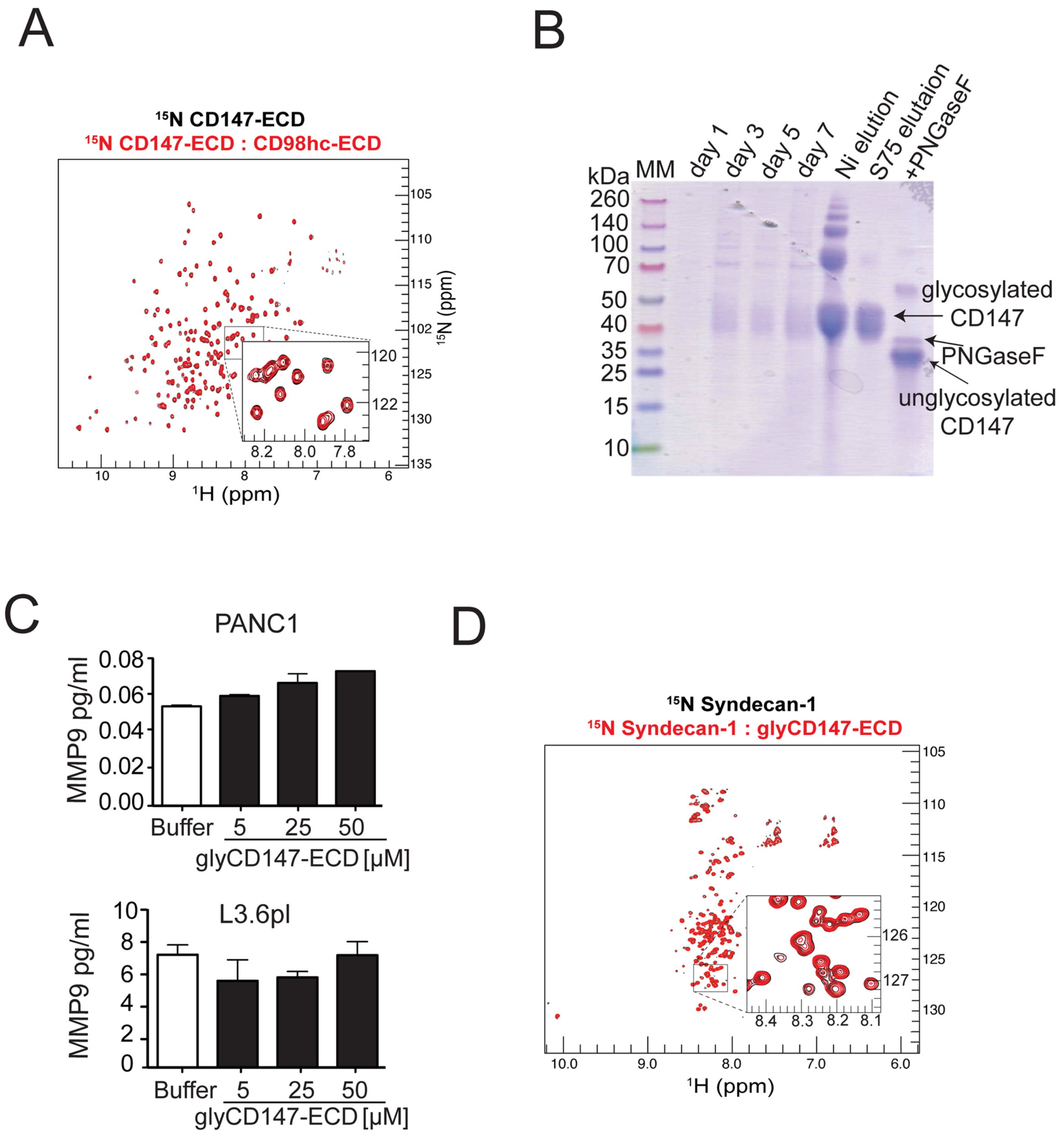

Supplementary Figure 7: CD147 ectodomain exhibits little to no activity and does not mediate its interactions. A. CD147 ectodomain does not interact with CD98hc ectodomain. ^{1}H/^{15}N HSQC NMR titration experiment for the uniformly ^{15}N labeled CD147-ECD and unlabeled CD98hc ectodomain (aa 212-630, CD98hc-ECD). Increased concentrations of unlabeled proteins were titrated into ^{15}N labeled protein (black - free protein). **B.** Glycosylated CD147 ectodomain (CD147-ECD^{gly}) can be produced and purified from mammalian cells. Coomassie stained SDS-PAGE gel showing different stages of CD147-ECD^{gly} expression (day 1-7) and elution from Ni-affinity and size exclusion (S75) columns. Last line shows PNGaseF treated purified CD147-ECD^{gly}. PNGaseF removes N-linked glycans as indicated by lower molecular weight observed. MM - molecular marker. **C.** CD147-ECD^{gly} exhibits little to no stimulatory activity in regard to PDAC cell lines. Indicated cells were stimulated with increasing concentrations of glycosylated CD147 ectodomain (CD147-ECD^{gly}) or buffer control for 24hrs in serum free media. Following treatment, conditioned media was collected and tested for MMP9 secretion via ELISA assay. Representative results are shown. Bars are ± SEM, n=2. **D.** CD147 ectodomain does not interact with some of its proposed partners. ^{1}H/^{15}N HSQC NMR titration experiment for the uniformly ^{15}N labeled Syndecan-1 and unlabeled glyCD147-ECD. Increased concentrations of unlabeled protein were titrated into ^{15}N labeled protein (black - free protein).

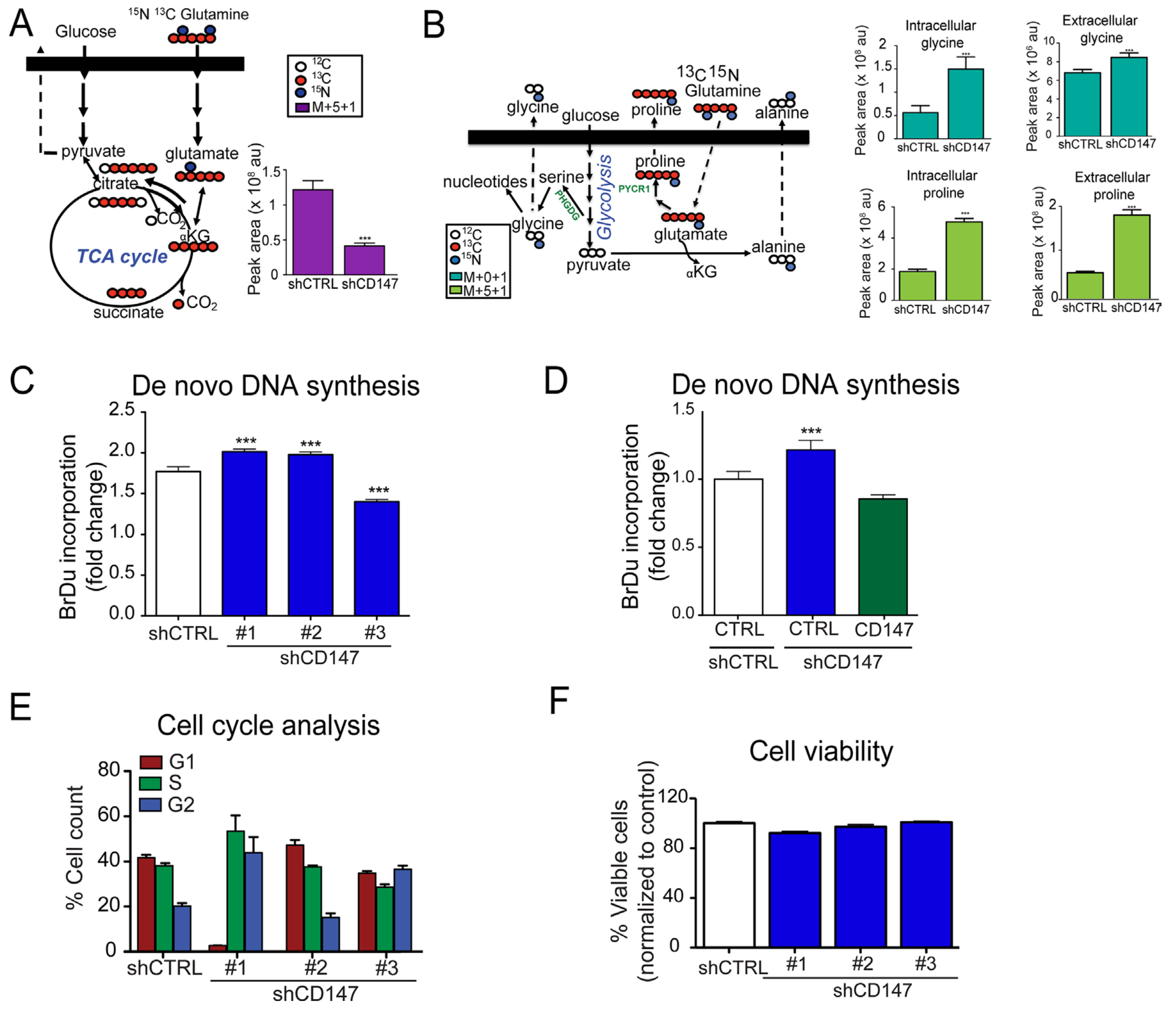

Supplementary Figure 8: Intracellular lactate accumulation and increased amino acid production, followed by concomitant proteome changes leads to a specific mechanism of cell adaptation to CD147 loss. A. and **B.** CD147 depleted cells increase their glutamine consumption and production of amino acids. Metabolite labeling experiments with $^{13}C_6$-glucose and $^{13}C_6$,$^{15}N_2$-glutamine in PANC1 shCTRL and shCD147 cells. See Methods for details. Proteins detected in SILAC experiments are in green. PYRC1 - pyrroline-5-carboxylase reductase, PHGDH - phsphoglycerate dehydrogenase, Bars are ± SEM, n=3, *p<0.05, **p<0.01 ***p<0.001. **C.** shCD147 cells exhibit a slight increase in nucleotide production. 5-bromo-2'-deoxyuridine (BrDu) incorporation was monitored using ELISA assay. Bars are ± SEM, n=3, ***p < 0.001. **D.** Re-expression of CD147 in shCD147 cells rescues the increased nucleotide production. BrDu incorporation was monitored using ELISA assay. Bars are ± SEM, n=3, ***p < 0.001. **E.** S/G2 cell cycle arrest in shCD147 PANC1 cells. Detailed analysis of cell cycle populations in the indicated cell lines from FACS cell cycle analysis in Figure 9E. **F.** CD147 knockdown does not significantly affect cell viability. Cellular viability was measured via Annexin V FACS assay.

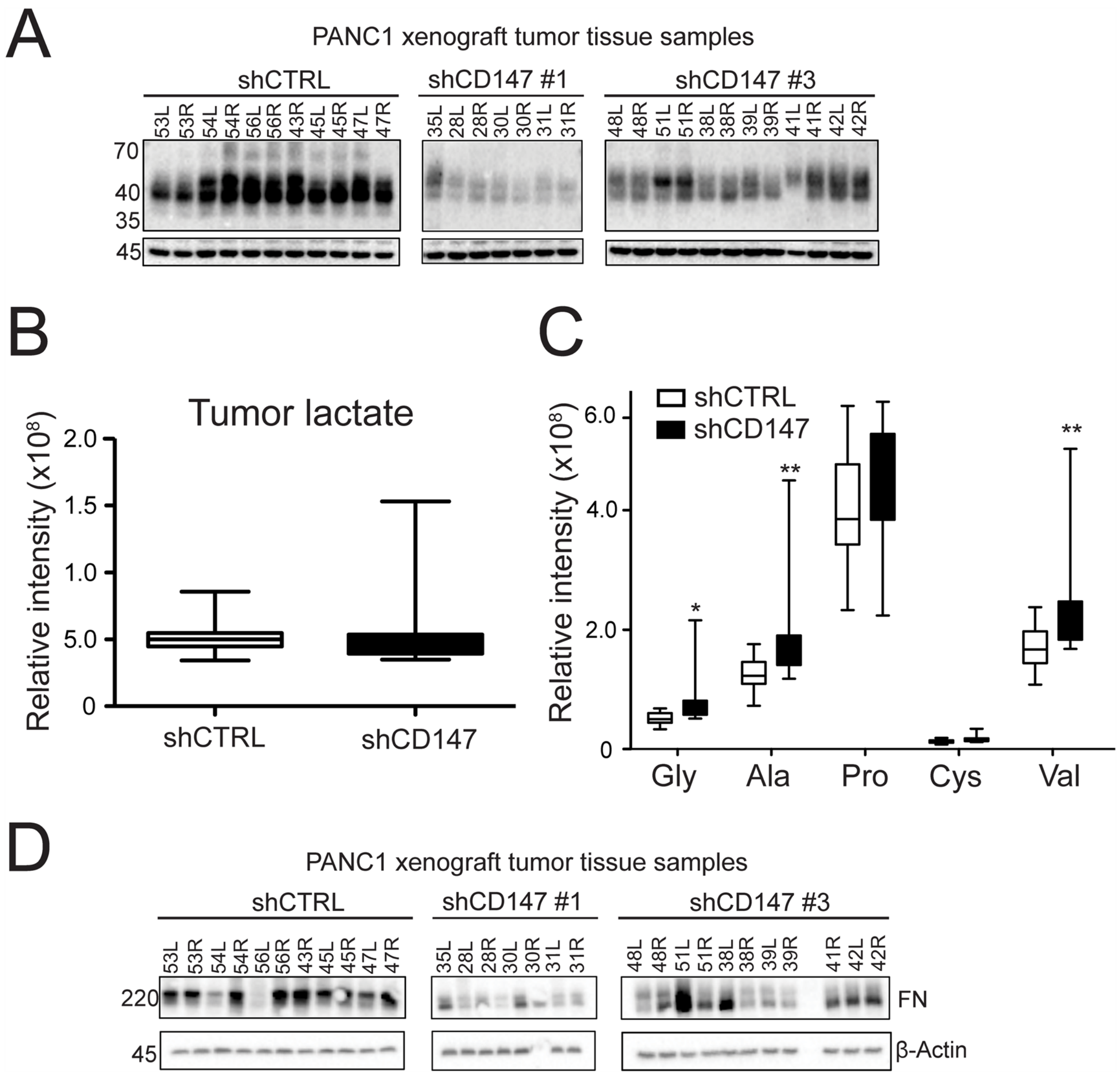

Supplementary Figure 9: The downregulation of CD147 expression is consistent with decreased tumor growth and diminished stromal environment leading to augmented amino acid production. A. CD147 downregulation does not persist over time for some of the knockdowns. Immunoblotting analysis of CD147 expression in tumor tissues. β-actin provided as a loading control. **B.** Total lactate level is not altered in CD147 depleted tumors. Metabolomics analysis of lactate levels in homogenized tumor tissues. Bars are ± SEM, n=15. **C.** Neutral amino acids are upregulated in shCD147 tissues. Metabolomics analysis of selected amino acid levels in homogenized tumor tissues. Bars are ± SEM, n=15, *p<0.05, **p<0.01. **D.** Decreased level of ECM marker (fibronectin, FN) in CD147 depleted tumors points to downregulated stromal environment. β-actin provided a loading control.

Supplementary Table 1: Proteins identified in cross-linking/pull-down MS experiments in PANC1 cells

See Supplementary File 1
