# Supplementary material for: CD147: a small molecule transporter ancillary protein at the crossroad of multiple hallmarks of cancer and metabolic reprogramming
Source: Oncotarget. 2016 Dec 27;8(4):6742–62. doi: 10.18632/oncotarget.14272 (PMC5341751; doi:10.18632/oncotarget.14272)
Supplement: Supplementary file 2 [file oncotarget-08-6742-s002.docx]

**Supplementry Table 1: Proteins identified in cross-linking/pull-down MS experiments in PANC1 cells.**

|  | **Exp. 1** | | | | **Exp. 2** | | | |
| --- | --- | --- | --- | --- | --- | --- | --- | --- |
| **Accession Number** | **GFP** | **CD147-GFP** | **GFP+DSS** | **CD147-GFP + DSS** | **GFP** | **CD147-GFP** | **GFP+DSS** | **CD147-GFP + DSS** |
| **P05023** | 0 | 0 | 0 | 28 | 0 | 0 | 0 | 43 |
| **P33527** | 0 | 0 | 1 | 45 | 0 | 1 | 1 | 51 |
| **P20020** | 0 | 0 | 0 | 28 | 0 | 1 | 0 | 34 |
| **O15427** | 0 | 6 | 0 | 7 | 2 | 7 | 0 | 8 |
| **Q6NZI2** | 0 | 0 | 1 | 15 | 0 | 0 | 2 | 16 |
| **Q01650** | 0 | 1 | 1 | 6 | 0 | 1 | 2 | 7 |
| **P02786** | 0 | 2 | 1 | 25 | 0 | 2 | 2 | 24 |
| **P05556** | 0 | 0 | 2 | 19 | 0 | 2 | 2 | 24 |
| **Q15758** | 1 | 2 | 2 | 11 | 2 | 2 | 2 | 12 |
| **Q8TCT9** | 0 | 1 | 0 | 8 | 0 | 0 | 0 | 9 |
| **Q96S97** | 0 | 1 | 1 | 5 | 0 | 2 | 2 | 6 |
| **O15031** | 0 | 0 | 0 | 22 | 0 | 0 | 0 | 26 |
| **P00533** | 0 | 1 | 2 | 26 | 1 | 3 | 2 | 28 |
| **P26006** | 0 | 0 | 0 | 14 | 0 | 0 | 0 | 19 |
| **P17301** | 0 | 0 | 0 | 21 | 0 | 0 | 1 | 21 |
| **Q9H5V8** | 0 | 6 | 0 | 9 | 0 | 13 | 0 | 12 |
| **Q9NZ01** | 1 | 2 | 2 | 5 | 2 | 2 | 2 | 5 |
| **P27797** | 0 | 2 | 0 | 9 | 0 | 0 | 1 | 10 |
| **Q03135** | 1 | 3 | 2 | 5 | 1 | 5 | 1 | 6 |
| **P13746** | 1 | 2 | 0 | 7 | 1 | 4 | 1 | 9 |
| **P61224** | 0 | 0 | 2 | 8 | 0 | 0 | 2 | 8 |
| **O43865** | 0 | 0 | 1 | 12 | 0 | 0 | 1 | 12 |
| **P39656** | 0 | 3 | 2 | 8 | 1 | 1 | 1 | 7 |
| **Q9UIW2** | 0 | 0 | 0 | 14 | 0 | 0 | 0 | 23 |
| **Q9UBV2** | 0 | 0 | 0 | 14 | 0 | 0 | 0 | 12 |
| **Q9Y666** | 0 | 0 | 0 | 12 | 0 | 0 | 0 | 17 |
| **Q99808** | 0 | 0 | 0 | 8 | 0 | 0 | 0 | 8 |
| **P50443** | 0 | 0 | 0 | 7 | 0 | 0 | 0 | 10 |
| **Q9Y6M7** | 0 | 0 | 0 | 13 | 0 | 0 | 1 | 18 |
| **P32004** | 0 | 0 | 0 | 17 | 0 | 0 | 0 | 15 |
| **O00214** | 0 | 6 | 0 | 8 | 0 | 7 | 0 | 10 |
| **Q9BRK3** | 0 | 0 | 0 | 11 | 0 | 1 | 0 | 13 |
| **P08581** | 0 | 1 | 0 | 8 | 0 | 7 | 0 | 17 |
| **P04899** | 0 | 1 | 1 | 9 | 1 | 1 | 1 | 9 |
| **P46977** | 0 | 2 | 2 | 9 | 1 | 0 | 0 | 4 |
| **P48960** | 0 | 0 | 0 | 10 | 0 | 1 | 0 | 13 |
| **Q9UGT4** | 0 | 0 | 0 | 9 | 0 | 0 | 0 | 11 |
| **Q13308** | 0 | 0 | 0 | 15 | 0 | 0 | 0 | 21 |
| **P36021** | 0 | 0 | 0 | 4 | 0 | 1 | 0 | 3 |
| **P04920** | 0 | 0 | 0 | 15 | 0 | 0 | 0 | 25 |
| **Q9NZW5** | 0 | 0 | 0 | 9 | 0 | 0 | 0 | 9 |
| **P42677** | 2 | 2 | 2 | 3 | 1 | 3 | 1 | 3 |
| **P14314** | 0 | 0 | 0 | 11 | 0 | 0 | 1 | 12 |
| **O60488** | 0 | 2 | 1 | 7 | 0 | 2 | 1 | 10 |
| **P21589** | 0 | 0 | 0 | 8 | 0 | 5 | 0 | 13 |
| **P54760** | 0 | 0 | 0 | 13 | 0 | 0 | 0 | 24 |
| **P48509** | 0 | 0 | 0 | 6 | 0 | 0 | 0 | 6 |
| **O14672** | 0 | 0 | 0 | 12 | 0 | 0 | 0 | 12 |
| **Q12797** | 0 | 0 | 0 | 12 | 0 | 1 | 0 | 3 |
| **P53985** | 0 | 0 | 0 | 5 | 0 | 0 | 0 | 5 |
| **Q9Y6M5** | 0 | 0 | 0 | 10 | 0 | 0 | 0 | 10 |
| **Q9BVK6** | 0 | 1 | 0 | 5 | 0 | 1 | 0 | 4 |
| **O95573** | 0 | 1 | 2 | 8 | 1 | 4 | 1 | 5 |
| **P49257** | 0 | 1 | 0 | 7 | 0 | 0 | 0 | 3 |
| **P60953** | 0 | 0 | 1 | 4 | 1 | 0 | 1 | 6 |
| **Q15005** | 0 | 1 | 1 | 4 | 0 | 1 | 0 | 3 |
| **Q8TCJ2** | 0 | 2 | 0 | 10 | 0 | 0 | 0 | 4 |
| **Q99805** | 0 | 0 | 0 | 6 | 0 | 0 | 0 | 7 |
| **O15126** | 0 | 0 | 0 | 5 | 0 | 0 | 0 | 9 |
| **P06756** | 0 | 0 | 0 | 15 | 0 | 0 | 0 | 9 |
| **O14828** | 0 | 0 | 0 | 4 | 0 | 2 | 1 | 4 |
| **Q9H3N1** | 0 | 0 | 0 | 5 | 0 | 1 | 0 | 7 |
| **P11717** | 0 | 0 | 1 | 13 | 0 | 0 | 0 | 7 |
| **P61586** | 1 | 0 | 0 | 5 | 2 | 2 | 2 | 5 |
| **Q9P035** | 0 | 0 | 0 | 8 | 0 | 0 | 0 | 3 |
| **O15439** | 0 | 0 | 0 | 9 | 0 | 0 | 0 | 13 |
| **P13987** | 0 | 0 | 0 | 4 | 0 | 2 | 1 | 4 |
| **Q96DZ1** | 0 | 0 | 0 | 8 | 0 | 0 | 0 | 6 |
| **Q96S52** | 0 | 0 | 0 | 9 | 0 | 0 | 0 | 6 |
| **Q5JPE7** | 0 | 0 | 0 | 15 | 0 | 0 | 0 | 3 |
| **Q6YHK3** | 0 | 0 | 0 | 9 | 0 | 0 | 0 | 13 |
| **P35240** | 0 | 0 | 1 | 6 | 0 | 0 | 2 | 4 |
| **P23634** | 0 | 0 | 0 | 9 | 0 | 0 | 0 | 10 |
| **P08648** | 0 | 0 | 0 | 10 | 0 | 0 | 0 | 13 |
| **P61106** | 1 | 0 | 2 | 4 | 1 | 0 | 0 | 5 |
| **P16435** | 0 | 0 | 2 | 9 | 0 | 0 | 1 | 8 |
| **O00186** | 0 | 0 | 0 | 9 | 2 | 3 | 0 | 5 |
| **Q9H223** | 0 | 0 | 1 | 4 | 0 | 1 | 1 | 8 |
| **Q8N766** | 0 | 0 | 0 | 9 | 0 | 0 | 0 | 3 |
| **P61019** | 2 | 1 | 2 | 3 | 1 | 4 | 1 | 6 |
| **P05026** | 0 | 0 | 0 | 4 | 0 | 0 | 0 | 3 |
| **Q9NTJ5** | 0 | 0 | 0 | 9 | 0 | 0 | 0 | 8 |
| **Q03519** | 0 | 2 | 0 | 5 | 1 | 1 | 1 | 3 |
| **P54709^#^** | 0 | 0 | 0 | 4 | 0 | 0 | 0 | 8 |
| **O14786** | 0 | 0 | 0 | 10 | 0 | 0 | 0 | 12 |
| **Q8NE01** | 0 | 0 | 0 | 4 | 0 | 0 | 0 | 8 |
| **Q8WUM9** | 0 | 0 | 0 | 10 | 0 | 0 | 0 | 6 |
| **Q4KMQ2** | 0 | 0 | 0 | 5 | 0 | 0 | 0 | 11 |
| **Q12907** | 0 | 0 | 0 | 5 | 0 | 0 | 0 | 7 |
| **Q92692** | 0 | 0 | 0 | 7 | 0 | 0 | 0 | 9 |
| **Q14160** | 0 | 0 | 0 | 8 | 0 | 0 | 0 | 14 |
| **Q8NF37** | 0 | 1 | 2 | 4 | 1 | 3 | 0 | 4 |
| **Q7L9B9** | 0 | 0 | 0 | 4 | 0 | 0 | 0 | 9 |
| **Q9NYU2** | 0 | 0 | 0 | 8 | 1 | 0 | 0 | 5 |
| **P16144** | 0 | 0 | 0 | 8 | 0 | 0 | 0 | 6 |
| **Q9Y4P3** | 0 | 2 | 0 | 4 | 1 | 0 | 1 | 4 |
| **P32970** | 0 | 0 | 0 | 6 | 0 | 0 | 0 | 6 |
| **Q9Y624** | 0 | 0 | 0 | 7 | 0 | 0 | 0 | 8 |
| **Q9BQE5** | 0 | 0 | 0 | 5 | 0 | 0 | 1 | 3 |
| **Q15599** | 0 | 1 | 0 | 5 | 0 | 0 | 0 | 7 |
| **P63000** | 0 | 0 | 0 | 5 | 0 | 0 | 1 | 5 |
| **Q03518** | 0 | 2 | 0 | 4 | 1 | 0 | 0 | 4 |
| **Q9H0X4** | 0 | 0 | 0 | 4 | 0 | 0 | 0 | 8 |
| **Q8TCZ2** | 0 | 0 | 0 | 3 | 0 | 0 | 0 | 4 |
| **Q9BTV4** | 0 | 0 | 0 | 6 | 0 | 0 | 0 | 7 |
| **Q96BD0** | 0 | 0 | 0 | 5 | 0 | 0 | 0 | 6 |
| **P43007** | 0 | 0 | 0 | 5 | 0 | 0 | 0 | 8 |
| **P53794** | 0 | 0 | 0 | 5 | 0 | 0 | 0 | 6 |
| **Q12959** | 0 | 0 | 0 | 6 | 0 | 0 | 0 | 5 |
| **P55085** | 0 | 0 | 0 | 5 | 0 | 0 | 0 | 5 |
| **P41440** | 0 | 0 | 0 | 5 | 0 | 0 | 0 | 6 |
| **P08754** | 0 | 0 | 1 | 5 | 0 | 0 | 0 | 5 |
| **Q92643** | 0 | 1 | 0 | 5 | 0 | 0 | 0 | 3 |
| **Q9HDC9** | 0 | 0 | 0 | 4 | 0 | 0 | 0 | 4 |
| **P30519** | 0 | 0 | 0 | 4 | 0 | 0 | 0 | 4 |
| **O15260** | 0 | 0 | 1 | 5 | 0 | 0 | 1 | 3 |
| **Q13641** | 0 | 0 | 0 | 3 | 0 | 0 | 0 | 6 |
| **Q658P3** | 0 | 0 | 0 | 4 | 0 | 0 | 0 | 5 |
| **P23229** | 0 | 0 | 0 | 7 | 0 | 0 | 0 | 7 |
| **Q13443** | 0 | 0 | 0 | 4 | 0 | 0 | 0 | 8 |
| **O00264** | 0 | 0 | 0 | 4 | 0 | 0 | 0 | 4 |
| **Q04771** | 0 | 0 | 0 | 5 | 0 | 0 | 0 | 4 |
| **P01130** | 0 | 0 | 0 | 7 | 0 | 0 | 0 | 7 |
| **O15438** | 0 | 0 | 0 | 6 | 0 | 0 | 0 | 7 |
| **Q13740** | 0 | 0 | 0 | 5 | 0 | 0 | 0 | 8 |
| **Q12846** | 0 | 0 | 0 | 4 | 0 | 0 | 0 | 5 |
| **Q9NZN4** | 0 | 0 | 2 | 4 | 0 | 0 | 0 | 7 |
| **P07099** | 0 | 0 | 0 | 5 | 0 | 0 | 0 | 3 |
| **P55011** | 0 | 0 | 0 | 7 | 0 | 0 | 0 | 7 |
| **O60245** | 0 | 0 | 0 | 8 | 0 | 0 | 0 | 6 |
| **O95297** | 0 | 0 | 0 | 3 | 0 | 0 | 0 | 4 |
| **Q9C0B5** | 0 | 0 | 0 | 4 | 0 | 0 | 0 | 4 |
| **P15529** | 0 | 0 | 0 | 3 | 0 | 0 | 0 | 3 |
| **P23458** | 0 | 0 | 0 | 7 | 0 | 0 | 0 | 4 |
| **O14493** | 0 | 0 | 0 | 3 | 0 | 0 | 0 | 4 |
| **P29323** | 1 | 0 | 0 | 4 | 0 | 0 | 0 | 5 |
| **Q969X5** | 0 | 0 | 1 | 4 | 0 | 1 | 1 | 3 |
| **Q8NBJ5** | 0 | 0 | 1 | 4 | 1 | 0 | 0 | 3 |
| **Q15006** | 0 | 0 | 1 | 4 | 0 | 0 | 1 | 4 |
| **Q9Y320** | 0 | 0 | 0 | 4 | 0 | 0 | 0 | 3 |
| **Q8IXU6** | 0 | 0 | 0 | 3 | 0 | 0 | 0 | 4 |
| **P48060** | 0 | 0 | 0 | 3 | 0 | 0 | 0 | 6 |
| **O00592** | 0 | 0 | 0 | 3 | 0 | 0 | 0 | 5 |
| **Q8TDW0** | 0 | 0 | 0 | 4 | 0 | 0 | 0 | 3 |
| **O15127** | 0 | 0 | 0 | 3 | 0 | 0 | 0 | 3 |
| **P30825** | 0 | 0 | 0 | 4 | 0 | 0 | 0 | 5 |
| **A7MBM2** | 0 | 0 | 0 | 4 | 0 | 0 | 0 | 3 |
| **Q9Y639** | 0 | 0 | 0 | 3 | 0 | 0 | 0 | 3 |
| **Q9Y289** | 0 | 0 | 0 | 3 | 0 | 0 | 0 | 4 |
| **Q9NV96** | 0 | 0 | 0 | 5 | 0 | 0 | 0 | 3 |
| **O95858** | 0 | 0 | 0 | 3 | 0 | 0 | 0 | 3 |
| **Q86X29** | 0 | 0 | 0 | 4 | 0 | 0 | 0 | 4 |
| **Q8N8Z6** | 0 | 0 | 0 | 4 | 0 | 0 | 0 | 3 |
| **P08195** | 1 | 13 | 2 | 25 | 9 | 20 | 4 | 27 |
| **P05362** | 0 | 1 | 3 | 18 | 2 | 5 | 5 | 21 |

Number of unique peptides are listed for each protein.
